# Combined LC–MS/MS and 16S rDNA analysis on mice under high temperature and humidity and Herb Yinchen protection mechanism

**DOI:** 10.1038/s41598-021-84694-9

**Published:** 2021-03-03

**Authors:** Yao Wang, Jiayi Chen, Jianbang Tang, Jiedong Xiao, Yuhua Zheng, Liting Tang, Huanhuan Luo

**Affiliations:** 1 School of Basic Medicine, Guangzhou University of Chinese Medicine, Guangzhou, 510006 Guangdong Province China; 2 School of Medical Information Engineering, Guangzhou University of Chinese Medicine, Guangzhou, 510006 Guangdong Province China; 3 Zhongshan Hospital of Chinese Medicine, No.3 Kangxin road, Xi district, Zhongshan City, Guangdong Province China; 4 Department of Endocrinology, Affiliated Hospital 1, Guangzhou University of Traditional Chinese Medicine , Guangzhou, 510405 Guangdong China

**Keywords:** Microbiology, Environmental sciences, Health care

## Abstract

With increased global warming, the impact of high temperature and humidity (HTH) on human health is increasing. Traditional Chinese medicine describes the Herb Yinchen as a remedy for reducing heat and eliminating dampness. This study focused on the impact of HTH conditions on mice and the potential protective effect of Herb Yinchen. Five male Balb/c mouse groups included two normal control groups, two HTH-exposed groups, and one Yinchen-treated group. For either three or ten days, normal and HTH-exposed mice were housed under normal or HTH (33 ± 2 °C,85% relative humidity) conditions, respectively. Yinchen-treated mice, housed under HTH conditions, received the Herb Yinchen decoction for three days. Metabolite profiles of plasma and liver samples from each group were analyzed using LC–MS/MS. Fecal DNA was extracted for 16S rDNA analysis to evaluate the intestinal microbiome. Spearman correlation analysis was performed on metabolites, bacteria, and bile acids that differed between the groups. We found that HTH altered the host metabolite profiles and reduced microbial diversity, causing intestinal microbiome imbalance. Interestingly, Herb Yinchen treatment improved HTH-mediated changes of the metabolite profiles and the intestinal microbiome, restoring them to values observed in normal controls. In conclusion, our study reveals that HTH causes intestinal bacterial disturbances and metabolic disorders in normal mice, while Herb Yinchen could afford protection against such changes.

## Introduction

Since the effects of global warming are becoming more serious, research efforts in assessing their impact on public health are substantially increasing worldwide. Epidemiological studies have shown that global warming has caused an increase in heat-related mortality in many countries^1^. Therefore, health problems caused by rising temperatures have become a serious public health concern^2^. Heat affects humans by a complex mechanism, involving interactions among temperature, radiation, wind, and humidity^3^. Meanwhile, humidity is also related to many human diseases. A previous study showed that the state of water in the atmosphere affects the core temperature and low hydration levels of the body and further affects heart function^4^. A study in western Sicily revealed a mixed relationship between relative humidity and angina pectoris^5^. Yang et al. found a positive correlation between relative humidity and hypertensive uremia^6^. Some studies suggest that long-term interactions between high temperature and humidity (HTH) are causing heat waves^7^ that can trigger public health emergencies. Therefore, it is important to study the effects of HTH on the body.

Herb Yinchen is the dry aerial part of *Artemisia scoparza Waldst. et Kit.* or *Artemisia capillaris Thunb.* It was first recorded in the Shen Nong Ben Cao Jing, a Chinese book on medicinal plants. It has a bitter flavor and is considered to have a cold nature in traditional Chinese medicine (CM). It mainly manifests its therapeutic actions in the spleen, gastric, liver, and gallbladder meridians. In CM theory, it has the effect of clearing heat, eliminating dampness, and protecting the liver function to provide relief from jaundice. Studies have shown that the ingredients contained in Herb Yinchen. exert anti-inflammatory effects through multiple inflammatory pathways or directly inhibit pain perception pathways (such as sodium/calcium influx)^8^. The extract of Herb Yinchen. is also reported to have a significant anti-fibrotic effect^9^ and a protective effect on alcohol-induced liver injury^10^. Herb Yinchen. plays a choleretic role by enhancing liver cell function, promoting its regeneration, and increasing excretion of bile acids (BA), phospholipids, and cholesterol in the liver^11^*.* Herb Yinchen. and its active ingredients can inhibit or kill a variety of viruses such as herpes simplex virus, poliovirus, influenza virus, hepatitis virus, human immunodeficiency virus, and severe acute respiratory syndrome (SARS) virus^12,13^. In this study, we used mice with altered bacterial flora and metabolic disorders induced by HTH to study the effects of HTH on intestinal microbiota, BA, and plasma metabolites and the protective effect of Herb Yinchen.The analysis included 16S rDNA gene sequencing, along with untargeted and targeted metabolomics method based on liquid chromatograph-mass spectrometer/mass spectrometer (LC–MS/MS). The results of our comprehensive study revealed the effects of the treatment with Herb Yinchen. on the metabolism in mice subjected to HTH.

## Results

### Effects of HTH on the plasma metabolite profile of mice

**Figure 1:**
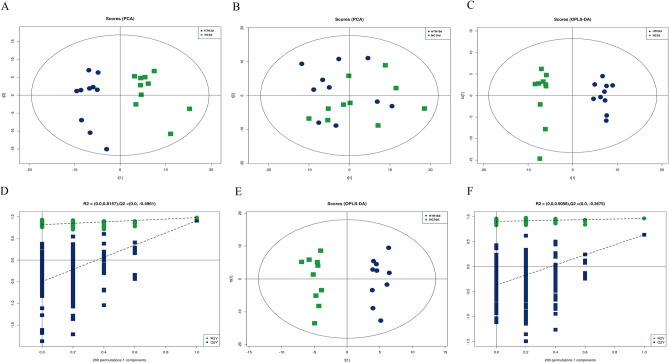
PCA scatter plot of the metabolite profile. The HTH3d group is separated from the NC3d (**A**), HTH10d and NC10d (**B**) components. OPLS-DA analyzes the metabolite profile. The OPLS-DA score graph (**C**) and OPLS-DA substitution test graph (**D**) show a good distinction between the HTH3d group and the NC3d group, R2X = 0.311, R2Y = 0.978, Q2 = 0.907, *p* < 0.05. The OPLS-DA score graph (**E**) and OPLS-DA substitution test graph (**F**) show a good distinction between the HTH10d and NC10d groups, R2X = 0.439, R2Y = 0.965, Q2 = 0.638, *p* < 0.05.

**Figure 2:**
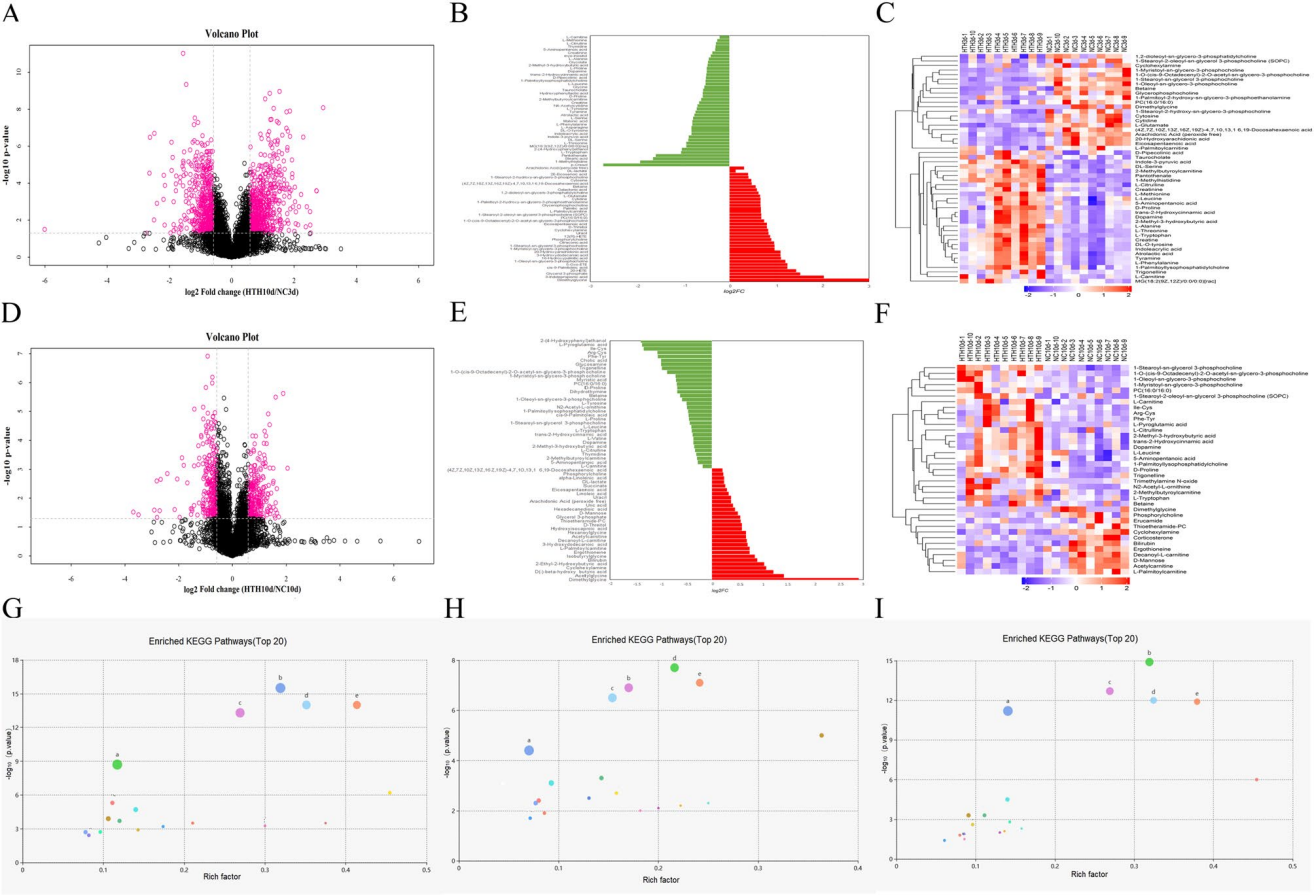
Screening and changing characteristics of various metabolites in plasma exposed to HTH environment at different times. The volcano graph shows the difference in metabolites of HTH3d_vs_NC3d (**A**) and HTH10d_vs_NC10d (**D**). The red dots in the figure are the metabolites with FC > 1.5 and *p* value < 0.05, which are the differential metabolites screened by univariate statistical analysis. Linear discriminant analysis (LDA) analyzes the size of the differential metabolites between HTH3d_vs_NC3d (**B**) and HTH10d_vs_NC10d (**E**). Hierarchical clustering heat map analysis of differential metabolites between HTH3d_vs_NC3d (**C**) and HTH10d_vs_NC10d (**F**). Each column represents a sample, and each row represents a metabolite. The color of each part corresponds to the concentration value of each metabolite calculated by the peak area normalization method (red, up; blue, down). (**G**, **H**, I): KO enrichment analysis bubble chart of metabolic pathways: a. ABC transporters; b. Protein digestion and absorption; c. Aminoacyl-tRNA biosynthesis, d. Central carbon metabolism in cancer, e. Mineral absorption. G: HTH3d_vs_NC3d group; H: HTH10d_vs_NC10d group; I: HTH10d_vs_HTH3d group.

To study the metabolic changes caused by exposure of mice to the HTH environment, we investigated the status of plasma metabolites in male Balb/c mice maintained under different conditions. Specifically, three and ten days after initiating the experiment, we performed LC–MS/MS analysis to compare the plasma metabolite profiles of mice kept under normal control conditions (groups NC3d and NC10d) with those exposed to the HTH conditions (groups HTH3d and HTH10d). As shown in the principal component analysis (PCA) diagram, the metabolites of the HTH3d and NC3d groups, and the HTH10d and NC10d groups were well separated (Fig. 1A,B). The orthogonal partial least squares discrimination analysis (OPLS-DA) model was used to further analyze the compounds that caused the differences between the different treatment groups. The OPLS-DA score indicated that the HTH3d group and the NC3d, HTH10d, and NC10d groups were scattered in two different areas. Goodness of fit and predictability (HTH3d vs. NC3d group: R2X = 0.311, R2Y = 0.978, Q2 = 0.907, *p* < 0.005; HTH10d vs. NC10d group: R2X = 0.439, R2Y = 0.965, Q2 = 0.638, *p* < 0.005) showed that the OPLS-DA model had a satisfactory fit and good predictive ability (Fig. 1C–F). According to the variable weights (Variable Importance for the Projection; VIP) obtained by the OPLS-DA model, the influence intensity and explanatory power of the expression patterns of each metabolite on the classification and discrimination of each group of samples were measured, and the differential metabolites with biological significance were selected. The metabolites that showed both multi-dimensional statistical analysis VIP > 1 and univariate statistical analysis *p* value < 0.05 were considered as significantly different metabolites, while those with VIP > 1 and 0.05 < *p* value < 0.1 were regarded as differential metabolites. In the HTH3d model, 50 of the detected plasma metabolites changed significantly; of these, 29 and 21 were upregulated and downregulated, respectively (Fig. 2A–C, Supplementary material 1). In the HTH10d model, 37 detected plasma metabolites showed significant changes (Fig. 2D–F). Furthermore, 25 and 12 species of intestinal bacteria showed increased and decreased abundance, respectively (Supplementary material 1). To further determine the relevant metabolic pathways involved in HTH3d, different metabolites were subjected to the “Kyoto Encyclopedia of Genes and Genomes” (KEGG) analysis to identify associated metabolic pathways; the results are shown in Fig. 2G–I. The key metabolic pathways enriched by HTH3d and HTH10d were found to be the same; these were ABC transporters; Protein digestion and absorption; Aminoacyl-tRNA biosynthesis, Central carbon metabolism in cancer, and Mineral absorption. Considering the time factor, we choose HTH3d for further research.

### Effects of HTH on the intestinal microbiome

**Figure 3:**
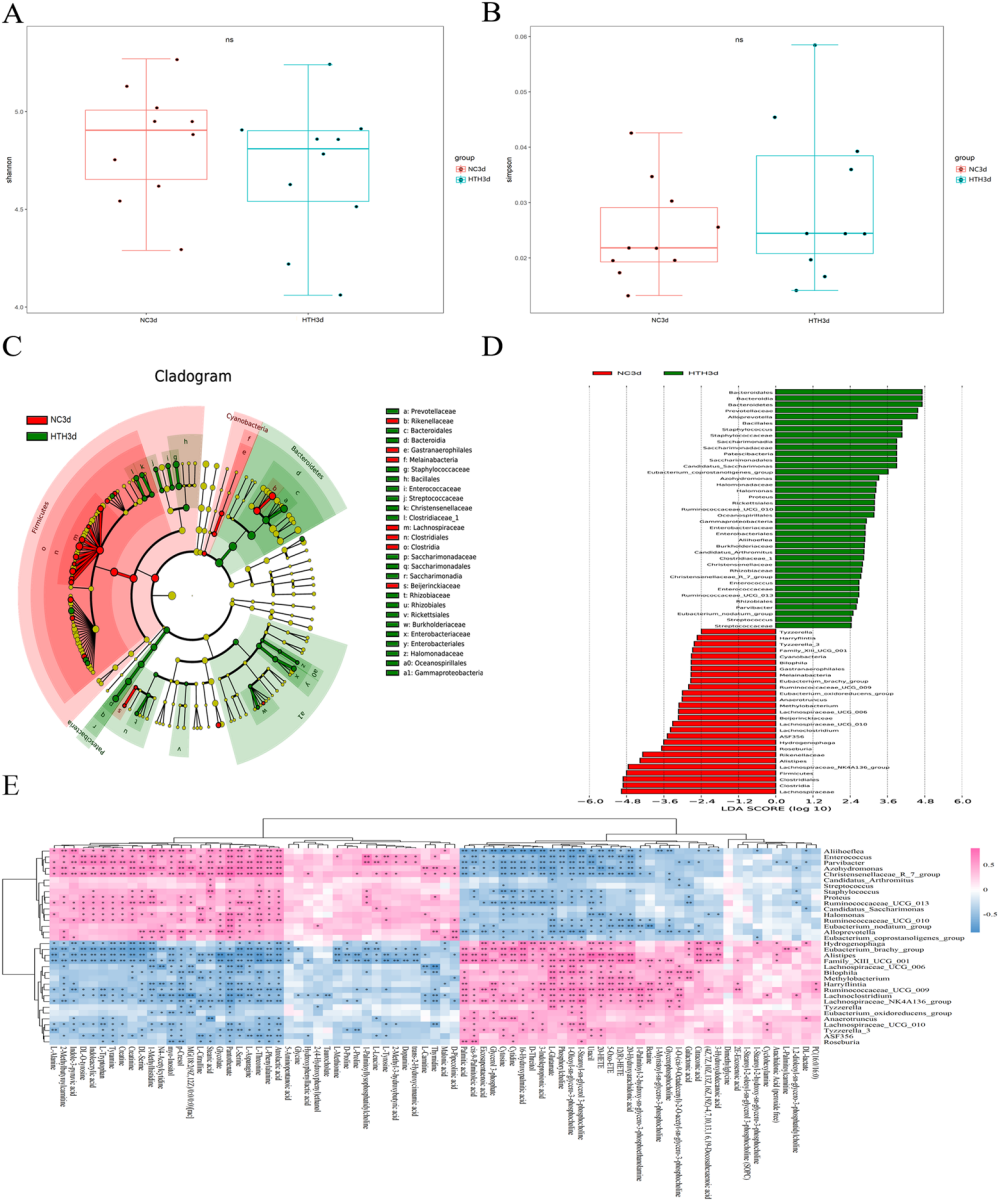
Alpha diversity of bacterial communities. Use box plots to visualize estimates of bacterial diversity (**A** and **B**). The boxplot shows the median, quartile, minimum, and maximum observations. Wilcoxon rank-sum test was performed to determine the statistical significance of the alpha diversity analysis. (**C** and **D**) LEFSe analysis of intestinal flora. The branch chart shows the differences in the fecal taxa. The letters correspond to taxonomic units with rich differences. The red node indicates the microbial group that plays an important role in the red group, the green node indicates the microbial group that plays an important role in the green group, and the yellow node indicates the microbial group that does not play an important role in both groups. The histogram represents the significant difference in abundance of LDA scores between bacteria (LDA > 2) compared groups, expressed in different colors. The taxa are displayed (LDA > 2). LEFSe: linear discriminant effect size; LDA: linear discriminant score. (**E**) The relationship between different triggering bacteria and plasma metabolites in different groups was expressed by a heat map, r > 0.5. r > 0 means positive correlation, expressed in red; r < 0 means negative correlation, expressed in blue. The darker the color, the stronger the correlation. *p* Value reflects the significant level of correlation, 0.01 < *p* value < 0.05, expressed by *, *p* value < 0.01, expressed by **.

Considering the relationship between the intestinal microbiome and metabolic status, we examined the effect of HTH on the gut bacteria in mice. 16S rDNA gene sequencing was used to evaluate changes in the intestinal microbiome triggered by exposure to HTH. First, alpha diversity metrics were used to calculate community diversity and richness. As shown in Fig. 3A,B, the Shannon Index and Simpson Index indicated that the HTH environment led to a significant reduction in the intestinal microbiome diversity of the HTH3d group compared to that of the NC3d group. We then used the linear discriminant analysis effect size (LEfSe) method (Fig. 3C) and the linear discriminant analysis (LDA) scores (Fig. 3D) to assess the differences in the distribution of dominant bacteria between the HTH3d and NC3d groups. LEfSe analysis is mainly used for high-dimensional biomarker discovery and identification of two or more genomic features. In this study, the LEfSe analysis was used with the Kruskal–Wallis test and paired Wilcoxon rank-sum test to detect significant differences in bacterial abundance and characteristics of the groups. Finally, LDA was carried out to estimate the impact of the abundance of each component (species) on the difference. The LEfSe analysis found that the following bacteria were more abundant in the HTH3d group than in the NC3d group: *Bacteroidales* at the order level; *Staphylococcaceae* at the family level; *Staphylococcus*, *Prevotella*, *Alloprevotella*, *Aliihoeflea*, *Azohydromonas*, and *Halomonas* at the genus level.

### Correlation between changes in plasma metabolites and the intestinal microbiome

We next investigated whether the changes in the intestinal microbiome and the metabolite spectrum were correlated. We used the Spearman correlation coefficient analysis to evaluate the differences in intestinal microbiota and plasma metabolites between the different groups. The analysis detected a significant correlation between changes in the gut microbiome and the metabolite profile (Fig. 3E), which included the following examples: the level of indole-3-pyruvic acid was positively correlated with the relative abundance of *Aliihoeflea*; the atrolactic acid and L-phenylalanine levels were positively correlated with the relative abundance of *Aliihoeflea* and *Azohydromonas*; the pantothenate and 2-methylbutyroylcarnitine levels were positively correlated with the relative abundance of *Alloprevotella*, *Aliihoeflea*, *Azohydromonas*, and *Halomonas*; the tyramine and creatinine levels were positively correlated with the relative abundance of *Aliihoeflea*, *Azohydromonas*, and *Halomonas.* Overall, these results indicated that changes in the intestinal microbiome were related to changes in the metabolite profile, and, moreover, there could be an interaction between these two factors with a direct effect on the metabolism in mice.

### Effect of HTH on BA

**Figure 4:**
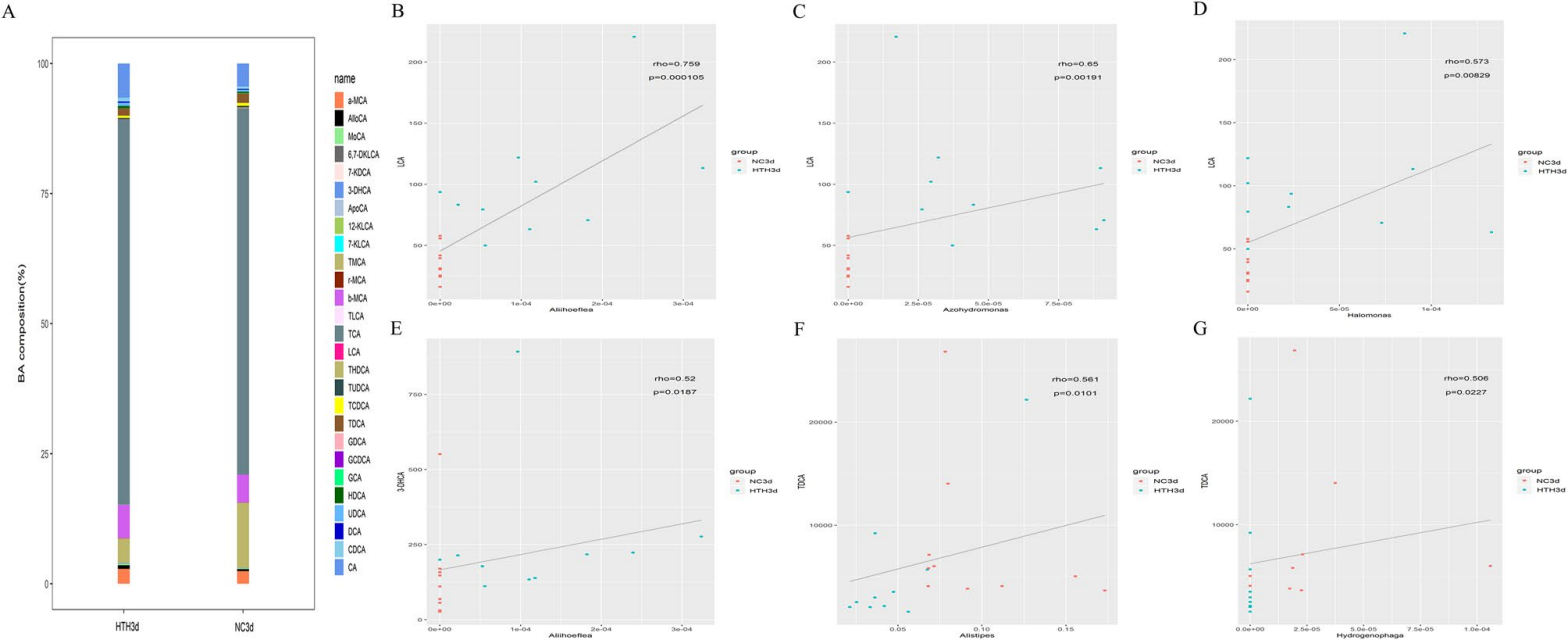
Generate a histogram of the composition of the bile acid pool based on the detection results of bile acids (**A**) to visually show the proportion of various bile acids and a higher proportion of bile acids. (**B**–**G**): The scatterplot illustrates the statistical correlation (*p* < 0.05) between the changed relative abundance of intestinal bacteria in mice and altered mouse liver bile acids.

As gut bacteria can participate in the formation of BA, we evaluated the effect of HTH-induced changes in gut bacteria on the composition of liver BA (Fig. 4A). The most common BAs in mouse liver biles are taurocholic acid (TCA) and tauromuricholic acid (TMCA).

### Association between BA and the intestinal microbiome

The intestinal microbiome regulated by the intestinal environment produces biologically active metabolites, such as short-chain fatty acids and BAs. The microbiota and BAs in the intestinal cavity have mutually dependent effects. Therefore, we examined bacterial group distribution in the feces and liver BAs. *Aliihoeflea* (r = 0.759, *p* = 0.000105), *Azohydromonas* (r = 0.65, *p* = 0.00191), and *Halomonas* (r = 0.583, *p* = 0.00829) were found to have a strong positive correlation with LCA (Fig. 4B–D). A strong positive correlation was also found between *Aliihoeflea* and 3-DHCA (r = 0.52, *p* = 0.0187) (Fig. 4E). *Alistipes* (r = 0.561, *p* = 0.0101), *Hydrogenophaga*, and taurodeoxycholic acid (TDCA) (r = 0.506, *p* = 0.0227) were also positively correlated (Fig. 4F,G).

### Association between liver BA and plasma metabolites

**Figure 5:**
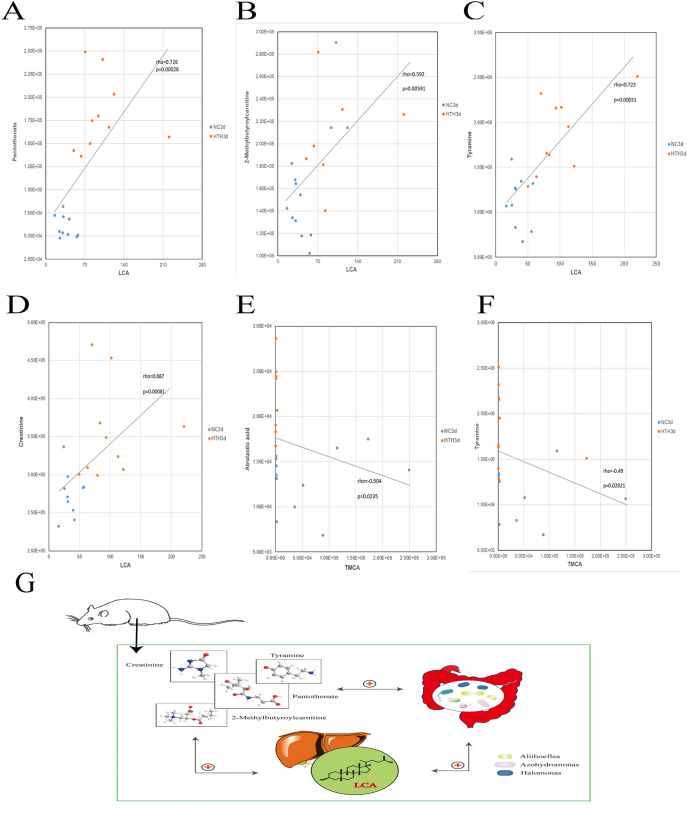
(**A**–**F**): The scatterplot illustrates the statistical association (*p* < 0.05) of altered mouse liver bile acids with some typical plasma metabolites. (**G**) Diagram of the interaction between plasma metabolites, intestinal microorganisms, and bile acids. “ + ”Stands for positive correlation. Two-way arrows represent interactions.

BAs in the gastrointestinal tract can alter the circulating metabolites, thereby affecting glucose, amino acid synthesis, and lipid metabolism. These metabolic effects may occur directly through the action of circulating BAs or indirectly by activation of receptors in the small or large intestine. Hence, we next analyzed the correlation between plasma metabolites and liver BA changes. LCA was positively correlated with pantothenate (r = 0.726, *p* = 0.00028), 2-methylbutyroylcarnitine (r = 0.592, *p* = 0.00591), tyramine (r = 0.723, *p* = 0.00031), and creatinine (r = 0.687, *p* = 0.00081) (Fig. 5A–D). TMCA was negatively correlated with atrolactic acid (r = 0.504, *p* = 0.0235) and tyramine (r = 0.49, *p* = 0.02821) (Fig. 5E,F). Combined with the above analysis, we found that: (1) pantothenate, 2-methylbutyroylcarnitine, tyramine, and creatinine are positively correlated with the levels of *Aliihoeflea*, *Azohydromonas*, and *Halomonas*; (2) LCA is positively correlated with the levels of *Aliihoeflea*, *Azohydromonas*, and *Halomonas*; and (3) LCA is related to pantothenate and 2-methylbutyroylcarnitine. Therefore, we speculate that the effect of HTH on mice may be achieved through the interaction of pantothenate, 2-methylbutyroylcarnitine, tyramine, creatinine, *Aliihoeflea*, *Azohydromonas*, *Halomonas*, and LCA (Fig. 5G).

### Effect of Herb Yinchen. on plasma metabolomics, intestinal microbiome, and BA in mice exposed to HTH

**Figure 6:**
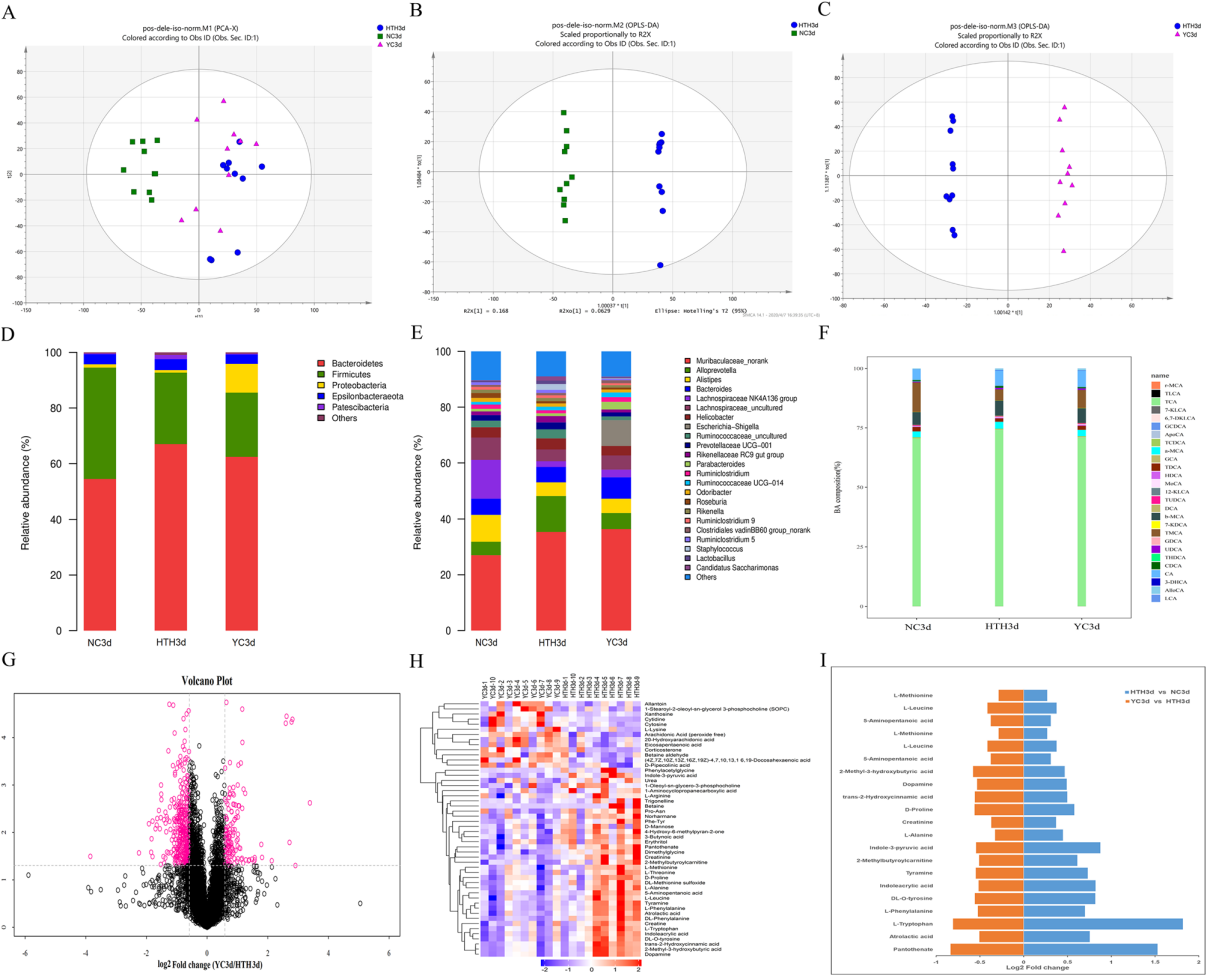
PCA scatter plot of the metabolite spectrum. (**A**) The NC group, HTH group, and YC group are separated from each other. OPLS-DA analyzes the metabolite profile. The OPLS-DA score chart (**B**) shows a good distinction between the HTH group and the NC group, R2X = 0.464, R2Y = 0.992, Q2 = 0.637, *p* < 0.05. OPLS-DA score chart (**C**) and a good distinction between the HTH and YC groups, R2X = 0.464, R2Y = 0.992, Q2 = 0.637, *p* < 0.05.Microbial species classification histogram (**D**, **E**): From phylum (**D**) and genus (**E**), respectively, it was shown that after administration of HTH, the intestinal bacterial of the HTH group showed significant changes; while that of the Herb Yinchen group tended to return to normal. Based on the results of bile acid detection, a histogram (**F**) of bile acid pool composition is generated to visually show the proportion of various bile acids and the higher proportion of bile acids. Screening and changing characteristics of various metabolites in plasma exposed to HTH environment at different times. The volcano graph shows the difference between metabolites in YC3d_vs_HTH3d (**G**). The red dots in the figure are the metabolites with FC > 1.5 and *p* value < 0.05, that is, the differential metabolites screened by univariate statistical analysis. Hierarchical clustering heat map analysis of differential metabolites between YC3d_vs_HTH3d (**H**). Each column represents a sample, and each row represents a metabolite. The color of each part corresponds to the concentration value of each metabolite calculated by the peak area normalization method (red, up; blue, down). The bidirectional histogram (**I**) shows metabolites with a fold change > 1 in the HTH3d_vs_NC3d group, and metabolites with a fold change < 1 in the YC3d_vs_HTH3d group.

According to the CM theory, Herb Yinchen. has the effects of clearing heat and eliminating dampness. Therefore, we studied the protective effect of Herb Yinchen. on metabolic disorders in mice exposed to the HTH conditions (YC3d group).In this research, one of the major ingredients of Herb Yinchen is chlorogenic acid (Supplementary Figure 1). First, we carried out principal component analysis (PCA) to evaluate the effects of Herb Yinchen. on plasma metabolites in HTH mice. The metabolic state of the HTH3d group differed significantly from that of the NC3d group (Fig. 6A–C). Further, the distance between the YC3d and the NC3d group was shorter than that between the HTH3d and the NC3d group. Thus, Herb Yinchen. could alleviate HTH-induced changes in the plasma metabolite profile of mice. A comparison of the bacterial species distribution indicated that the YC3d group had a greatly increased abundance of intestinal bacteria at the phylum and genus levels, compared to that in the HTH3d group, and there was a trend toward the NC3d group (Fig. 6D,E). Furthermore, a histogram of the detected BA pool was generated to visually display the proportions of various BAs. We found that the proportions of BAs varied significantly between the NC3d group and the HTH3d group. Interestingly, the YC3d group displayed a greatly improved BA profile compared with that of the HTH3d group, with a trend toward the NC3d group (Fig. 6F). In summary, these results indicated that Herb Yinchen. had a protective effect on HTH-induced metabolic disorders in mice.

### Effects of Herb Yinchen. on plasma metabolites in HTH mice

To further study the mechanism underlying the protective effect of Herb Yinchen. on HTH mice, we identified metabolites with fold change ≥ 1 in the comparison between HTH3d vs. NC3d group and YC3d vs HTH3d group (Figs. 2C, 6G,H and Supplementary material 2). Based on our analysis, we identified that pantothenate, 2-methylbutyroylcarnitine, tyramine, and creatinine were significantly increased in the HTH3d group, but decreased upon treatment with Herb Yinchen. treatment (Fig. 6I).

### Correlation between effects of Herb Yinchen. on plasma metabolites, intestinal microbiome, and BAs in HTH mice

**Figure 7:**
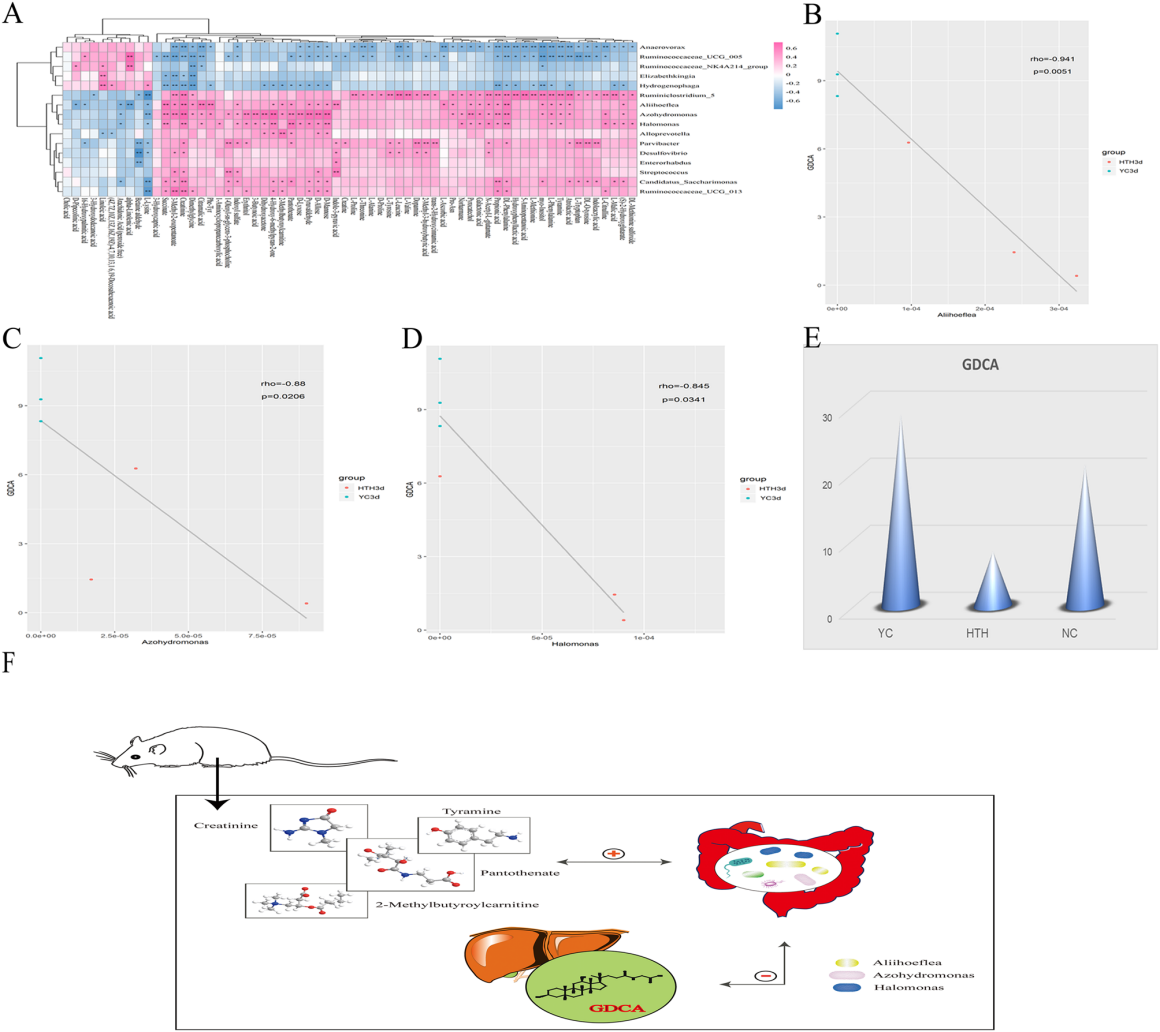
(**A**) The relationship between different triggering bacteria and plasma metabolites in different groups was expressed by a heat map, r > 0.5. r > 0 means positive correlation, expressed in red; r < 0 means negative correlation, expressed in blue. The darker the color, the stronger the correlation. *p* Value reflects the significant level of correlation, 0.01 < *p* value < 0.05, expressed by *, *p* value < 0.01, expressed by **. (**B**, **C**, **D**) The scatterplot illustrates the statistical association of altered mouse liver bile acids with some typical plasma metabolites (*p* < 0.05). (**E**) The histogram shows the change of bile acid GDCA. (**F**) Diagram of the interaction between plasma metabolites, intestinal microorganisms, and bile acids. “ + ” Stands for positive correlation. Two-way arrows represent interactions.

We identified similarities and differences in the bacteria profiles at the genus level and the metabolite profiles by performing a Spearman (r > 0.5) correlation hierarchical clustering analysis using significant differential bacteria and metabolites from the different groups. The analysis results are presented as a hierarchical clustering heat map (Fig. 7A). Based on the data in Figs. 3E and 7, we identified the intestinal bacteria that were related to the levels of pantothenate, 2-methylbutyroylcarnitine, tyramine, and creatinine. In the comparison of the HTH3d vs. the NC3d group, we found that *Aliihoeflea*, *Azohydromonas*, and *Halomonas* were correlated with the levels of pantothenate, 2-methylbutyroylcarnitine, tyramine, and creatinine. These correlations were also observed in the comparison of the YC3d vs. the HTH3d group. Furthermore, in the YC3d-vs.-HTH3d comparison, the glycodeoxycholic acid (GDCA) levels were negatively correlated with the abundance of *Aliihoeflea*, *Azohydromonas*, and *Halomonas* (Fig. 7B–D). Moreover, the GDCA levels were significantly decreased in the HTH3d group but increased in the YC3d group due to the treatment with Yinchen (Fig. 7E). Therefore, we speculate that the protection of Herb Yinchen. on HTH3d mice may be achieved through the interaction of pantothenate, 2-methylbutyroylcarnitine, tyramine, and creatinine and *Aliihoeflea*, *Azohydromonas*, and *Halomonas* to regulate GDCA(Fig. 7F).

## Discussion

With growing industrialization and urbanization, global warming has become a serious global concern. Long-term climate warming may cause the incidence of extreme heat waves to increase worldwide^14^. HTH significantly affects the generation of heatwaves^15,16^. According to the second law of thermodynamics, if the ambient temperature is higher than the optimal human core temperature (approximately 37 °C), the human body will not be able to dissipate heat^17^. Sweating is the main process through which the body releases heat. If the relative humidity in the environment is high, the efficiency of sweating is reduced, resulting in heat accumulation in the body^18^, increasing morbidity and mortality^19^. HTH can increase the levels of TNF-α, IL-1β, IL-6, and IL-17 in rat serum and activate the NF-kB pathway to cause inflammation^20^. It can also increase the content of CD14 and TLR4 in mouse lung tissue^21^.

The underlying mechanism of the effect of HTH on mice is not yet fully understood, and research using advanced technology is still required to clarify the exact molecular mechanism(s). This study was focused on the adverse impact of HTH exposure on mice. We aimed to identify HTH-induced changes in the plasma metabolite profiles, fecal microbiota, and BA content in mice, along with the protective effects of Herb Yinchen. against these changes. This may be the first study to use 16S rDNA gene sequencing and LC–MS/MS-based metabolomics analysis to examine the effect of HTH on mice metabolism. We exposed mice to HTH for 3 or 10 days and observed the consequent effects on plasma metabolite profile, intestinal microbiota, and BA in mice. We observed that HTH3d compared with NC3d; HTH10d compared with NC10d mice have significant differences. Considering the time factor, we choose HTH3d for further research.

Our subsequent analysis revealed that HTH exposure significantly changes the plasma metabolic profile in mice. The levels of dimethylglycine, betaine, cytidine, cytosine, L-glutamate, eicosapentaenoic acid, arachidonic acid, 20-hydroxyarachidonic acid, and other metabolites were significantly reduced in the HTH group compared to those in the NC group, while l-leucine, alanine, l-tryptophan, 1-methylhistidine, 2-methylbutyroylcarnitine, pantothenate, tyramine, l-phenylalanine, and indole-3-pyruvic acid levels showed an upward trend. The levels of 2-methylbutyroylcarnitine affect lipid and fat metabolism^22^. Tyramine is considered the most toxic biological amine and can induce migraine^23^ or hypertension^24^. Tyramine also has cytotoxic effects on human intestinal epithelial cell lines^25^. Besides, high temperature is known to increase the concentration of tyramine^22^, which is consistent with our findings.

Pantothenate is the water-soluble vitamin B5^26^, hich is a precursor in the synthesis of coenzyme A (CoA). The synthesis of citric acid by oxaloacetic acid and acetyl-CoA is an important regulatory step of the tricarboxylic acid cycle. The HTH-induced decreased levels of glycine and glutamate and the increased levels of pantothenate indicated that HTH accelerated the tricarboxylic acid cycle and energy expenditure, further suggesting that energy metabolism was disrupted by HTH exposure. The KEGG analysis also revealed that the differential metabolites were involved in energy metabolism (Central carbon metabolism in cancer, Mineral absorption) and protein production and metabolism [ATP-binding cassette (ABC) transporters, Protein digestion, and absorption, Aminoacyl-tRNA biosynthesis]. Central carbon metabolism in cancer affects the morphology of bacteria in some ways, such as by affecting the cell cycle process^27^ and lipid synthesis^28^. Metabolites associated with the central carbon metabolism in cancer can enter the tricarboxylic acid cycle. The ABC transporter can actively transport many substrates and participate in cell metabolism and/or biomolecule transport^29^. At the same time, the ABC transporter is an outlet for fatty acids, cholesterol, peptides, and sterols, and plays a role in antioxidant stress response, detoxification, and antigen presentation^29^. combining these data, such metabolomics studies may partially reveal the mechanism of action of HTH on mice.

Intestinal bacteria also affect the intestinal function and immune system. Therefore, we performed 16S rDNA gene sequencing to identify intestinal bacteria associated with the response to HTH. The HTH group had a lower intestinal microbial diversity than the NC group, and the major differences were associated with Bacteroidetes (phylum), Staphylococcaceae (family), and Staphylococcus, Alloprevotella, Aliihoeflea, Azohydromonas, Prevotella, and Halomonas (genus). These results indicate that exposure to HTH could disrupt the intestinal microecological structure. The association of HTH exposure with intestinal malnutrition and enteritis in mice is already known^30^. The intestinal microbiota may play an intermediary role and increase the sensitivity of mice to HTH metabolism. Intestinal bacteria use the ingredients in the diet to produce energy and metabolites, many of which are absorbed into the bloodstream, where they can be further metabolized or affect the metabolism of the host. This indicates that the disturbance of the intestinal microbiome caused by HTH may be the etiological pathway leading to overall metabolic disorder in mice. Besides, bacteria in the HTH environment can affect the microenvironment in mice, which in turn affects the microbiome.

The altered plasma metabolites showed correlation with intestinal microbes. For example, pantothenate, 2-methylbutyroylcarnitine, tyramine, and creatinine were positively correlated with the relative abundance of *Aliihoeflea, Azohydromonas,* and *Halomonas.* Earlier studies reported that many pantothenate antimetabolites inhibit the growth of *Escherichia coli* and *Staphylococcus aureu*^26^. According to Gardini et al., various factors, such as temperature and microorganisms affect the tyramine content^31^. Most tyramine-producing bacteria are Gram-positive bacteria, including *enterococci, Lactobacillus* sp., *Staphylococcus aureus*, and *Lactococcus* sp.^32–35^. This is consistent with our findings. A recent study showed that creatinine could increase the mortality of patients with spontaneous bacterial peritonitis^36^. These results indicate that there may be a link between the destruction of the composition of the intestinal microbiota and the increase in diversity and development of metabolic homeostasis, which manifests as a metabolic disorder in mice.

BAs can regulate the homeostasis of intestinal bacteria by inhibiting bacterial growth and preventing bacteria from adhering to the top of the intestinal mucosa. BAs can also activate Farnesoid X receptor, maintain the homeostasis of intestinal flora, prevent bacterial migration, and enhance the defense function of the mucosal barrier^37^. Studies suggest that the BA content in the intestine and the excessively grown of intestinal bacteria may cause inflammatory diseases^38^. *Bacteroides fragilis*, *Bacteroides vulgaris*, *Listeria perfringens*, other *Listeria* sp., lactic acid bacteria, and *Bifidobacterium* can produce bile salt hydrolyzing enzymes in the intestine, hydrolyzing bound BAs into free BAs^39^. Deoxycholic acid can be produced by *Clostridium* sp. such as *Clostridium absonum* to produce UDCA^40^. LCA is the most hydrophobic and toxic secondary BA, produced by bacterial metabolism of chenodeoxycholic acid or UDCA in the colon^40^. In this study, LCA was found to have correlations with *Aliihoeflea*, *Azohydromonas*, and *Halomonas*. LCA can cause cirrhosis^41^ and destroy placental function^42^. Based on the above, we believe that HTH changes the metabolic homeostasis of mice by altering the abundance of plasma metabolites (pantothenate, 2-methyl utyroylcarnitine, tyramine, creatinine), intestinal bacteria (*Aliihoeflea*, *Azohydromonas*, *Halomonas*), and BA (LCA) interaction.

In CM theory, Herb Yinchen. has a cooling effect, can eliminate dampness, and protect the liver function^43^. We found that Herb Yinchen. has a corrective effect on the metabolic bias of HTH-induced mouse plasma metabolites, intestinal microbes, and BA metabolism. Further, its therapeutic effect on HTH mice was found to occur via the interaction between pantothenate, 2-methylbutyroylcarnitine, tyramine, creatinine and *Aliihoeflea*, *Azohydromonas*, *Halomonas* and consequent regulation of the BA GDCA. Increased GDCA can inhibit the synthesis of BA^44^, and hence, a decrease in GDCA may cause cholestasis. HTH reduces GDCA levels in mice, increases LCA levels, and increases the risk of cholestasis. Previous studies have suggested that Herb Yinchen. can relax biliary tract muscle expansion, promote bile secretion, and increase the output of BAs and bilirubin in bile^45^. Herb Yinchen. Could also inhibit *Leptospirosis bomona* and SARS virus^46^. Furthermore, Herb Yinchen. and its components could inhibit glucose-6-phosphatase activity, thereby inhibiting gluconeogenesis in the liver and regulating glucose metabolism in the body^47^. After intervention with Herb Yinchen., GDCA and LCA levels were increased in HTH mice, which further proved its therapeutic effects. Thus, the protective effect of Herb Yinchen. on HTH mice may be achieved by regulating the interaction between intestinal bacteria and host metabolism to further regulate BAs.

This study has some limitations. First, this study did not determine whether there is a difference in the metabolic response to HTH between men and women. Second, it does not determine the order of interaction between plasma metabolites, intestinal microecology, and BAs. Besides, this study still lacks the relationship between metabolic changes and symptoms. Finally, the mechanism of action of Herb Yinchen. did not perfectly link with the mechanism of action of HTH, because this study did not find significant plasma metabolites and BAs that have similar correlation with HTH mice after Herb Yinchen. Future research should consider using sterile mice to study the effect of HTH environment on metabolism. In addition, evaluation of the physical condition of mice and high-throughput sequencing can be combined to further study the effect of HTH on mice.

## Conclusion

This study combined 16S rDNA gene sequencing and LC–MS/MS metabolomics to analyze the effect of HTH on the metabolism of mice and the protective effect of Herb Yinchen. Our results support the following conclusions. (1) The effect of HTH on the metabolism in mice is related to pathways associated with aminoacyl-tRNA biosynthesis, ABC transporter, protein digestion and absorption, mineral absorption, and central carbon metabolism in cancer. (2) The HTH-induced disturbance of the metabolism in mice occurs via interactions involving intestinal bacteria (*Aliihoeflea*, *Azohydromonas*, *Halomonas*), BA (LCA), and plasma metabolites (pantothenate, 2-methylbutyroylcarnitine, tyramine, creatinine). (3) Herb Yinchen. has a protective effect on HTH mice through interaction between plasma metabolites (pantothenate, 2-methylbutyroylcarnitine, tyramine, creatinine) and intestinal bacteria (*Aliihoeflea*, *Azohydromonas*, *Halomonas*) to regulate the production of BAs (GDCA).

## Materials and methods

### Materials

#### Experimental instruments

AB Triple TOF 6600 (Quadrupole Time-of-Flight) Mass Spectrometry (AB SCIEX, USA); Agilent 1290 Infinity LC Ultra-High Pressure Liquid Chromatography (equipped with Binary pump, Card-type flow cell, automatic sampler, UV detector; Agilent Technologies Inc, USA); HILIC: 2.1 mm × 100 mm ACQUIY UPLC BEH 1.7 µm column (Waters, USA); Eppendorf 5430R High-speed refrigerated centrifuge (Eppendorf, Germany); MP Fastprep-24 Automated Homogenizer MP (MP Biomedicals, USA); Scientz JY92-II Ultrasonic Liquid Processors (Ningbo Scientz Biotechnology Co., Ltd., China); Concetrator plus/Vacufuge (Eppendorf, Germany); Vortex (Shanghai QiTe Analytical Instrument Co., Ltd., China); Electronic balance (Metler-Toledo Instruments; AL104); Pipette (Eppendorf, Germany); IVC Independent Ventilation System (Suzhou Fengshi Laboratory Animal Equipment Co., Ltd., China); Yadu YC-D205 Ultrasonic humidifier (Beijing Yadu Environmental Technology Co., Ltd. , China); RXZ-158A-LED Artificial climate box (Ningbo Jiangnan Instrument Factory, China);Normal-temperature centrifuge (Thermo Fisher Scientific, USA); Low-temperature refrigerator (Haier Company, China); WIGGENS Vortex Shaker (WIGGENS, Germany); DK-8D Three Temperatures Three Controls Constant Temperature Water Bath (Shanghai Boxun Industrial Co., Ltd., China); Bio-rad T100 gradient PCR instrument (Bio-rad, USA); EPS 300 Electrophoresis Instrument (Tanon Science & Technology Co., Ltd. China); Tanon-2500 Gel Imager (Tanon Science & Technology Co., Ltd. China); Qubit@ 2.0 Fluorometer (Thermo Scientific,USA); Bioanalyzer 2100 system (Agilent, USA). 5500 QTRAP Mass Spectrometry (AB SCIEX, USA); Waters ACQUITY UPLC I-Class system (Waters; USA); HILIC: Waters, ACQUITY UPLC BEH C18 1.7 µm, 2.1 mm × 100 mm column; Prominence-I L 2030C series HPLC (Prominencei, LC-2030C, Tokyo, Japan); Agilent Zorbax Eclipse XDB-C18 column (250 × 4.6 mm i.d., 5 µm; Agilent, CA, USA).

#### Experimental reagent

CTAB (Solarbio C8440, USA) ; agarose gels (Biowest 111,860, Spain); Phusion High-Fidelity PCR Master Mix (New England Biolabs, UK); SYBR Green I(10,000 ×) (Solarbio SR4110, USA), 100 bp DNA Ladder(SolarbioM1200, USA); Qiagen Gel Extraction Kit (Qiagen 28704, Germany); TruSeq DNA PCR-Free Sample Preparation Kit (Illumina FC-121-3001, USA); 50 × TAE Buffer (ST716 Shanghai Beyotime Biotechnology Co., Ltd., China); phosphoric acid (3A, China); Chlorogenic acid (Macklin 60618068, China); 95% ethanol (Xinxiang Sanwei Disinfectant Co., Ltd., China); acetonitrile (Merck I592230123, Germany); CH3COONH4(Sigma70221, USA); NH3·H2O (Fluka, USA); Trifluoroacetic Acid(Sigma T6508, USA); Formic Acid (Fluka 06450, USA); Formic acid (Honeywell 94318, USA); Methanol (Fisher Chemical A452-4, USA); Isopropanol (Fisher Chemical A461-4, USA); Acetonitrile(Sigma, 34851-4L, USA); Bile acid standards were purchased from Sigma-Aldrich and Steraloids. The bile acid standards were 12-ketolithocholic acid(12-KLCA), 3-dehydrocholic acid\3-oxocholic acid(3-DHCA), 6,7-diketolithocholic acid(6,7-DKLCA), 7-ketodeoxycholic acid(7-KDCA), 7-Ketolithocholic acid(7-KLCA), Chenodeoxycholic acid (CDCA), Cholic acid (CA), Deoxycholic acid (DCA), Glycochenodeoxycholic acid(GCDCA), Glycocholic acid(GCA), Glycodeoxycholic acid(GDCA), Glycohyodeoxycholic acid (GHDCA), Glycolithocholic acid(GLCA), Glycoursodeoxycholic acid(GUDCA), Hyodeoxycholic acid(HDCA), Isolithocholic acid(isoLCA), Lithocholic acid(LCA), murocholic acid (Mo-CA), Taurochenodeoxycholic acid(TCDCA), Taurocholic acid(TCA), Taurodeoxycholic acid(TDCA), Taurohyodeoxycholic acid(THDCA), Taurolithocholic acid(TLCA), Ursodeoxycholic acid(UDCA), α-Muricholic acid(α-MCA), β-Muricholic acid(β-MCA), γ-muricholic acid\hyocholic acid(γ-MCA/HCA), ω-Muricholic acid(ω-MCA), Allocholic acid(AlloCA), Apocholic acid(ApCA), Tauro α-muricholic acid(α-TMCA), Tauro β-muricholic acid(β-TMCA), Tauro ω-muricholic acid(ω-TMCA), Taurohyocholic acid(THCA).

#### Animal

Sixth-six SPF-grade male Balb/c mice weighing 100 ± 10 g were purchased from Guangdong Medical Laboratory Animal Center (Foshan, China). Under the license number: SCXK (Guangdong) 2019–0035. The animals were fed adaptively for 1 week at 18–22 °C in a Specific Pathogen Free (SPF) animal laboratory, with access to food and water 24 h a day.

#### Experimental medication

Herb Yinchen is the dry aerial part of *Artemisia scoparza Waldst. et Kit.* or *Artemisia capillaris Thunb.* It was collected from Guangzhou University of Chinese Medicine and identified by Professor Luo Huanhuan, Guangzhou University of Chinese Medicine.

According to the 2015 edition of the Pharmacopoeia of the People's Republic of China, the clinical dosage of Herb Yinchen is 6–15 g. In this experiment, we take the middle value of 10 g^43^.

Herb Yinchen decoction extraction method: Herb Yinchen 1 kg, add 5L of water, boil 3 times, every half an hour, boil once every 30 min, filter out and store in a common container, and finally get 2.5L solution(0.4 g/ml) .

### Methods

#### Animal grouping and intervention administration

After one week of adaptive feeding, Balb/c mice were divided into five groups according to the principle of weight uniformity: two normal control groups (NC3d, NC10d), two high-temperature and high-humidity groups (HTH3d, HTH10d), and Herb Yinchen (YC), 20 mice in each group. The two HTH groups and YC groups were established by culturing in an HTH environment (T: 33 ± 2 °C, RH: 85%) for three and ten days respectively. The YC group was also given an intervention by Herb Yinchen Tonga for three days. In the Yc group, 0.4 μg/ml Herb Yinchen soup was administered. The dose is 0.5 ml/100 g body weight. The mice in the NC and HTH groups were treated with an equal amount of normal saline. Interventions were given by intragastric administration once a day for 3consecutive days.

#### Collection and treatment of samples

Twenty-four hours after the last gavage, stool samples from each group of mice were collected by abdominal compression, the mice were sacrificed, and the mouse plasma and liver were taken. Feces, plasma, and liver samples were stored in enzyme-free liquid nitrogen. After collection, the samples were transferred to a low-temperature refrigerator and immediately stored at − 80 °C.

#### LC–MS/MS analysis

Plasma samples set under 4 °C, 100 μL aliquots and 400 μL cold methanol/acetonitrile (1:1, v/v) were mixed and centrifuged for 20 min (14000 g, 4 °C) to remove the protein. A vacuum centrifuge dried and stored the supernatant. LC–MS/MS method analyzed the dissolving supernatant dissolved by 100 μL acetonitrile/water (1:1, v/v).

Samples were separated using an Agilent 1290 Infinity LC Ultra Performance Liquid Chromatography System (UHPLC) HILIC column; column temperature 25 °C; flow rate 0.3 mL/min; mobile phase composition A: water + 25 mM ammonium acetate + 25 mM ammonia, B: Acetonitrile; gradient elution procedure is as follows: 0–0.5 min, 95% B; 0.5–7 min, B linearly changes from 95 to 65%; 7–8 min, B linearly changes from 65 to 40%; 8–9 min, B maintained at 40%; 9–9.1 min, B linearly changed from 40 to 95%; 9.1–12 min, B maintained at 95%; samples throughout the analysis Place in 4 °C autosampler. To avoid the influence caused by the fluctuation of the signal detected by the instrument, continuous analysis of the samples is performed in a random order. QC samples are inserted into the sample queue to monitor and evaluate the stability of the system and the reliability of experimental data.

Electrospray ionization (ESI) positive and negative ion modes were used for detection. The samples were separated by UHPLC and analyzed by mass spectrometry using an Agilent 6550 mass spectrometer. The ESI source conditions are as follows: Gas Tem: 250 °C, Drying gas: 16 L/min, Nebulizer: 20 psig, Sheath gas Tem: 400 °C, Sheath Gas Flow: 12 L/min, Vcap: 3000 V, Nozzle voltage: 0 V. Fragment: 175 V, Mass Range: 50–1200, Acquisition rate: 4 Hz, cycle time: 250 ms.

After the samples were detected, the metabolites were identified using AB Triple TOF 6600 mass spectrometer, and the first and second spectra of QC samples were collected. The ESI source conditions are as follows: Ion Source Gas1 (Gas1): 40, Ion Source Gas2 (Gas2): 80, Curtain gas (CUR): 30, source temperature: 650 °C, IonSapary Voltage Floating (ISVF) ± 5000 V (plus or minus two) Mode); the secondary mass spectrum is obtained using information dependent acquisition (IDA), and the high sensitivity mode is used. Declustering potential (DP): ± 60 V (positive and negative modes), Collision Energy: 35 ± 15 eV, IDA settings are as follows Exclude isotopes within 4 Da, Candidate ions to monitor per cycle: 10. The data collection is segmented according to the mass range, 50–300, 290- 600, 590–900, 890–1200, thereby expanding the acquisition rate of the secondary spectrum, each method collects four replicates per segment. The collected data were used to identify the structure of metabolites using the self-built MetDDA and LipDDA methods, respectively.

#### HPLC chromatographic conditions

The chromatographic quantitative analysis was conducted on a Prominence-i L 2030C series HPLC system (Prominence-i, LC-2030C, Tokyo, Japan), equipped with a DAD (190–800 nm), a binary pump, and an auto sampler. An Agilent Zorbax Eclipse XDB-C18 column (250 × 4.6 mm, 5 µm; Agilent, CA, USA) was used for separation and maintained at 30 °C, and the column flow rate was 1.0 mL/min. The binary gradient phase consisted of 0.05% phosphoric acid in water (solvent A) and acetonitrile (solvent B) and used the following gradient for separation: 10% B at 0–20 min; 10–80% B at 20–30 min; 80% B at 30–60 min. The wavelength of the UV signal monitoring was selected as 327 nm according to pharmacopoeia, and the injection volume was 10.0 μL.

### 16S rDNA microbial community analysis

#### DNA extractions

Total genome DNA from samples was extracted using the CTAB/SDS method. DNA concentration and purity were monitored on 1% agarose gels. According to the concentration, DNA was diluted to 1 ng/μl using sterile water.

#### PCR amplification and 16S rDNA sequencing

The V3-V4 region of the prokaryotic (bacterial and archaeal) small subunit (16S) rDNA gene was amplified with slightly modified versions of primers 341F (5′- CCTAYGGGRBGCASCAG-3′) and 806R (5′- GGACTACNNGGGTATCTAAT-3′)^18^. 16S rDNA genes were amplified used the specific primer with the barcode. All PCR reactions were carried out in 30 μL reactions with 15 μL of Phusion High-Fidelity PCR Master Mix (New England Biolabs); 0.2 μM of forward and reverse primers, and about 10 ng template DNA. Thermal cycling consisted of initial denaturation at 98 °C for 1 min, followed by 30 cycles of denaturation at 98 °C for 10 s, annealing at 50 °C for 30 s, and elongation at 72 °C for 30 s. Finally 72 °C for 5 min. Mix the same volume of 1X loading buffer (contained SYB green) with PCR products and operate electrophoresis on 2% agarose gel for detection. Samples with a bright main strip between 400 and 450 bp were chosen for further experiments. PCR products were mixed in equidensity ratios. Then, mixture PCR products were purified with the GeneJET Gel Extraction Kit (Thermo Scientific). Sequencing libraries were generated using TruSeq DNA PCR-Free Sample Preparation Kit following the manufacturer’s recommendations and index codes were added. The library quality was assessed on the Qubit@ 2.0 Fluorometer (Thermo Scientific) and Agilent Bioanalyzer 2100 system. At last, the library was sequenced on an Illumina MiSeq and 250 bp paired-end reads were generated.

#### Bile acid detection

Liver samples(30 mg) were homogenized with 200 µl pre-cooled ultrapure water, add 800 µl, add 800 μL of pre-cooled methanol and 10 μL of internal standard, vortex, and precipitate the protein for 20 min at − 20 °C; Centrifuge at 14,000 rcf for 15 min at 4 °C and take the supernatant to dry under vacuum; add 100 μL methanol–water (1:1, v/v) for reconstitution. Centrifuge at 14,000 rcf for 15 min at 4 °C and take the supernatant for analysis. Take the standard and dilute it to a series of gradient concentration standard working solution with methanol aqueous solution, prepare the standard curve solution according to the above method, and establish the standard curve by the isotope internal standard method. Samples were analyzed using an Acquity UPLC system (Waters Ltd.) coupled online to 5500 QTRAP Mass Spectrometry (AB SCIEX, USA). The samples (2ul) were injected onto an ACQUITY UPLC BEH C18 1.7 μm, 2.1 mm × 100 mm column (Waters Ltd.). The samples were eluted at a flow rate of 250 μL/min with phase A (0.1% formic acid in water) and phase B (Methanol). The separation was performed as followed: linear gradient from 60 to 85% B (0–15 min), isocratic at 85% B (15–17 min), linear gradient from 85 to 60% B (17–17.1 min), and isocratic at 60% B (17.1–20 min). The column temperature was 45 °C. A QC sample is set for each set of experimental samples in the sample queue to test and evaluate the stability and repeatability of the system.

Samples were separated on Waters UPLC system. Mobile phase: 0.1% FA aqueous solution in phase A and methanol in phase B. The sample was placed in an 8 °C autosampler with a column temperature of 45 °C, a flow rate of 250 μL/min, and an injection volume of 2 μL. The relevant liquid phase gradient is as follows: 0–7 min, phase B linearly changes from 60 to 70%; 7–15 min, phase B linearly changes from 70 to 85%; 15–17 min, phase B is maintained at 85%; 17–17.1 min, the phase B changed linearly from 85 to 60%; 17.1–20 min, phase B was maintained at 60%. A QC sample is set for each set of experimental samples in the sample queue to test and evaluate the stability and repeatability of the system.

Mass spectrometry was performed as follows: source temperature: 550 °C; ion Source Gas1 (Gas1): 55; Ion Source Gas2 (Gas2): 55; Curtain gas (CUR): 40; ionSapary Voltage Floating (ISVF): − 4500 V; The MRM mode is used to detect the transitions to be measured. For information on the transitions of all bile acids, see Supplement Materials.

#### Statistical analysis

Samples were sequenced on an Illumina MiSeq platform. Paired-end reads were merged using FLASH^48^. Quality filtering on the raw tags was performed under specific filtering conditions to obtain high-quality, clean tags according to the Fast QC (V 0.10.1)^49^. Chimeric sequences were filtered using Useach software (10 version). Sequences with 97% similarity were assigned to the same operational taxonomic units (OTUs) by Usearch (10 version). Representative sequences were chosen for each OTU, and taxonomic data were then assigned to each representative sequence using the RDP (Ribosomal Database Project) classifier.

Sequences were processed with the software package of the QIIME (V1.9.0)^50^ toolkit. Taxonomy-based analyses were conducted by classifying each sequence using the SILVA database (https://www.arb-silva.de/). Alpha diversity, beta diversity, and rarefaction curve analyses were performed based on the relative OTU abundance table. Alpha diversity including Chao1, Shannon, Simpson, and Observed species, which was analyzed using Mothur. Principal coordinate analysis (PCoA) was performed using R software (R 3.4.2) and linear discriminant analysis (LDA) coupled with effect size measurement (LefSe) analysis was conducted online (http://huttenhower.sph.harvard.edu/galaxy/).

The original data collected by LC–MS/MS were processed by XCMS Online (https://xcmsonline.scripps.edu)^51^, which can provide a comprehensive method of data processing, including retention time correction and peak alignment. After the data pre-processing, the dataset was generated consisting of the m/z value, retention time, and the related peak areas and was imported into SIMCA-P software (14.1 Version, Umetrics AB, Sweden) for multivariate statistical analysis (PCA analysis, PLS-DA analysis, OPLS-DA). By using a combination of multivariate statistical analysis of OPLS-DA and univariate statistical analysis, differential metabolites were screened(VIP > 1, *p* < 005). Semi-quantitative analysis (heat map) and cluster analysis of potential biomarkers by Mev (version 4.9.0) software were performed. Finally, the metabolic pathway databases such as KEGG (http://www.genome.jp/kegg/)^52^ were used to perform enrichment analysis and network construction of related metabolic pathways.

The raw LC–MS/MS data were analyzed using the Multiquant Software to obtain calibration equations and the quantitative concentration of each BA in the samples. Differences in the BA measurements between the groups were analyzed using a Student’s t-test with *p* < 0.05 considered significant. The regression equation was derived from the standard curve, the relative deviation of the samples’ lower limit of quantification (LLOQ) was ≤ 20%, the relative deviation of the other concentrations and quality control relative standard deviation (QC RSD) was ≤ 30% and the square of the correlation coefficient R was > 0.99. The content of each test sample was calculated from the standard curve.

Spearman correlation analysis was used to analyze the correlation between fecal microflora and metabolomics. Using R software (version 3.3.1) to generate graphics, we get the results with *p* < 0.05, which are statistically significant.

One-way ANOVA was performed using the Social Science statistics Software Package (SPSS 22.0, Chicago, USA). The significance threshold of this experiment was *p* < 0.05. All statistical tests were performed using Graphpad Prism, version 8.0.

### Ethical approval

All experiments were approved by the Animal Experimental Ethical Inspection Form of Zhongshan Hospital of TCM (Protocol 2019018) and performed by the recommendations of the NIH Guide for the Care and Use of Laboratory Animals [National Research Council. Guide for the Care and Use of Laboratory Animals. (2011)]. The study was carried out in compliance with the ARRIVE guideline.

## Supplementary Information

Supplementary Information 1.

Supplementary Information 2.

Supplementary Information 3.

Supplementary Information 4.

Supplementary Information 5.

Supplementary Information 6.

Supplementary Information 7.
