# Supplementary figures and images for: Combined LC–MS/MS and 16S rDNA analysis on mice under high temperature and humidity and Herb Yinchen protection mechanism
Source: Sci Rep. 2021 Mar 3;11:5099. doi: 10.1038/s41598-021-84694-9 (PMC7930127; doi:10.1038/s41598-021-84694-9)

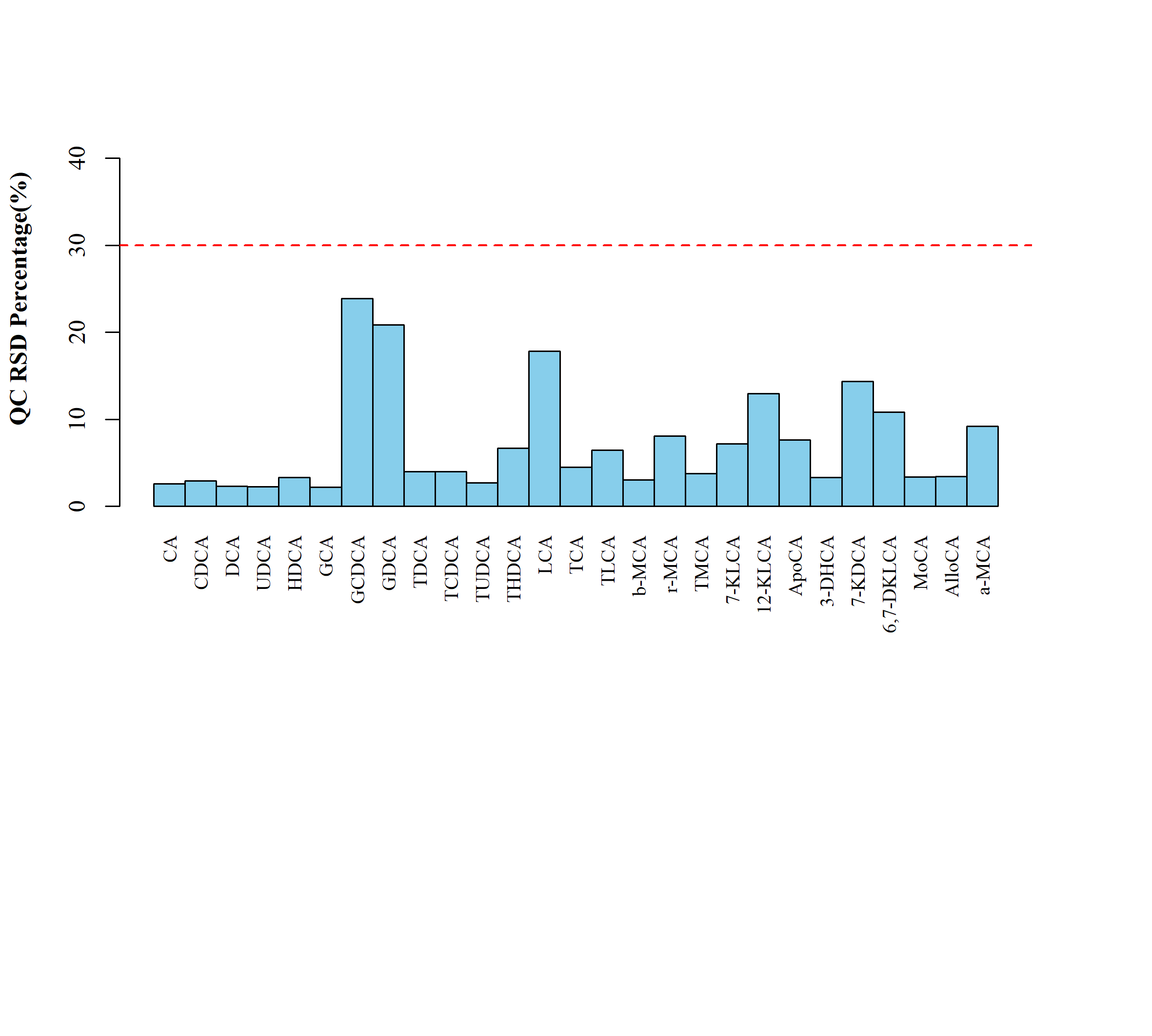

Supplement: Supplementary file 2 — Supplementary Information 2. [file 41598_2021_84694_MOESM2_ESM.tiff]

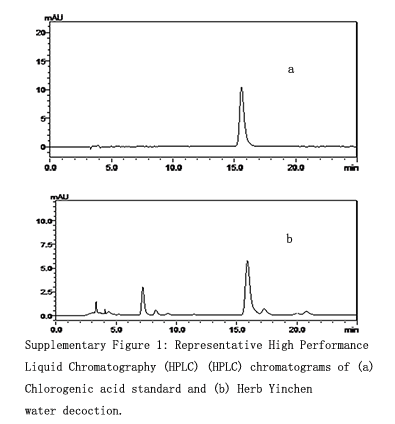

Supplement: Supplementary file 3 — Supplementary Information 3. [file 41598_2021_84694_MOESM3_ESM.tif]

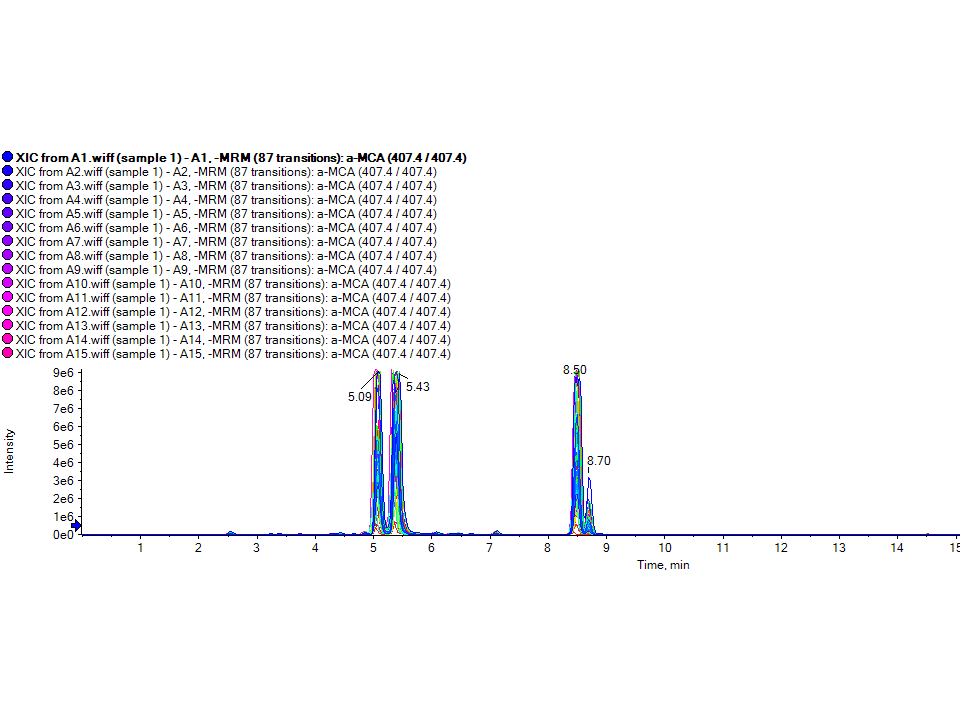

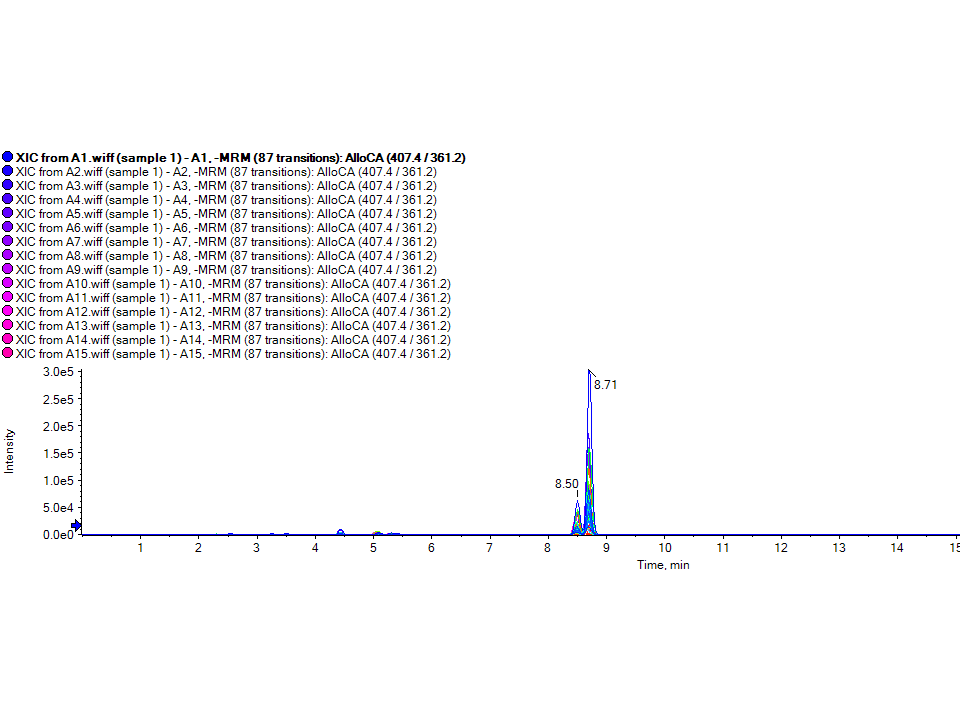

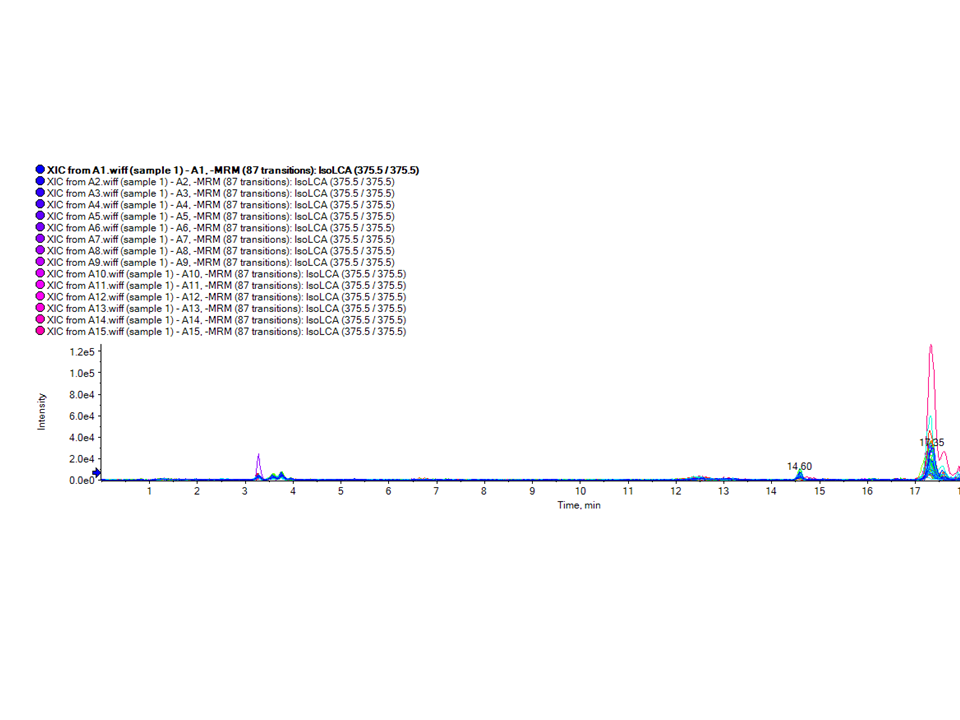

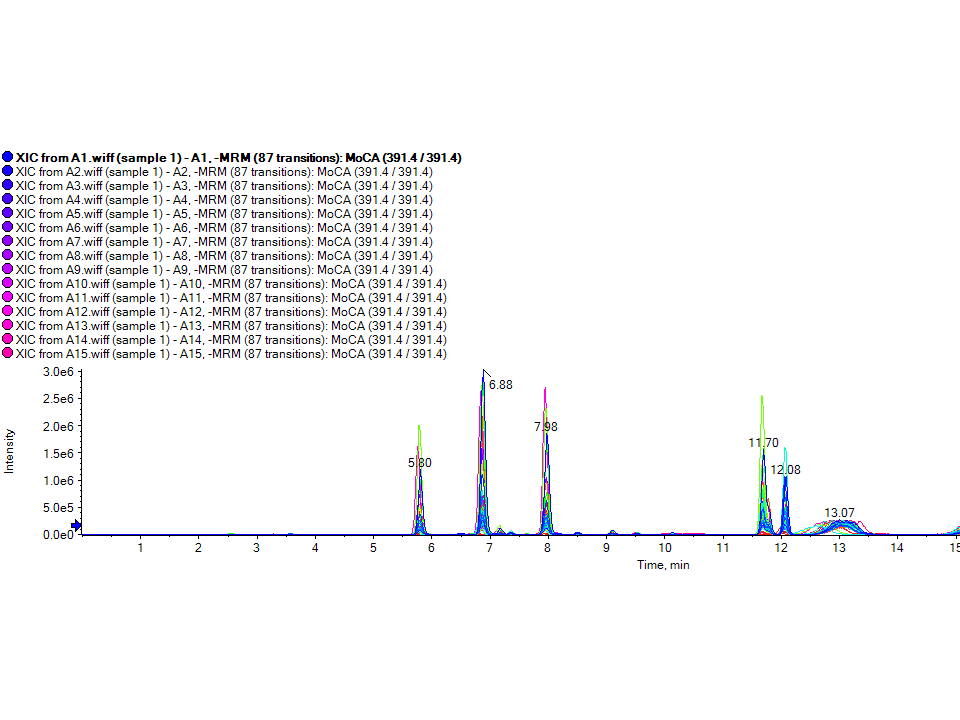

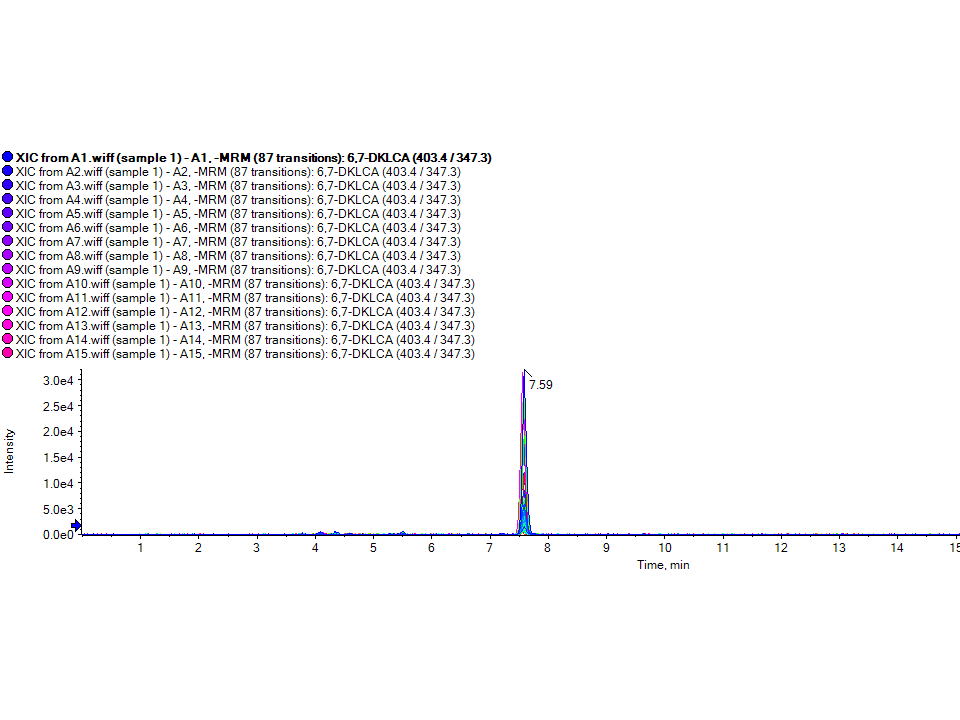

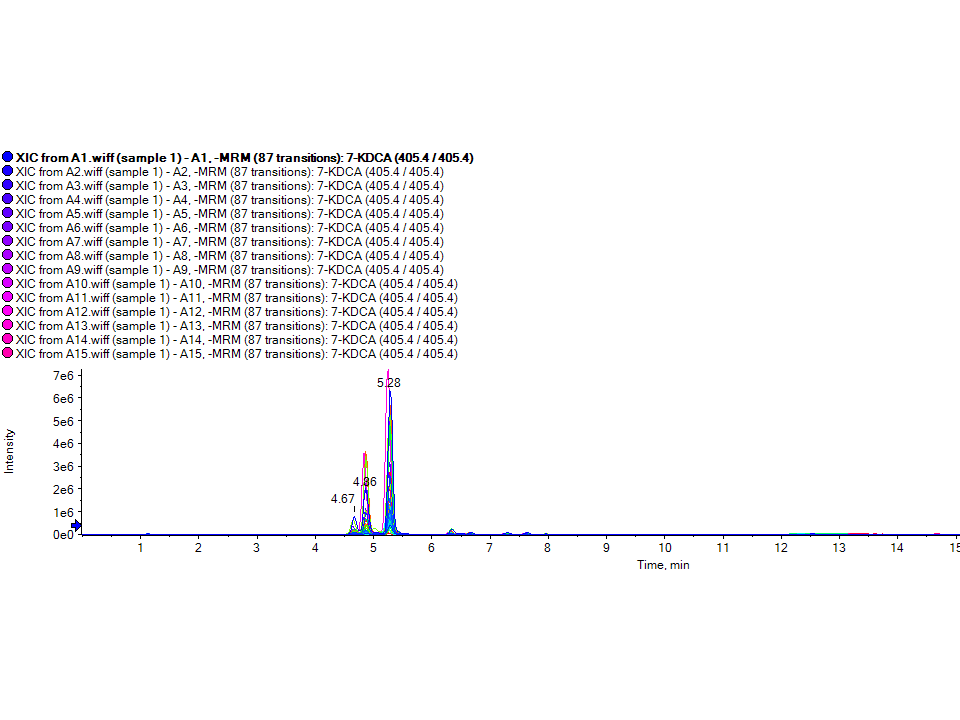

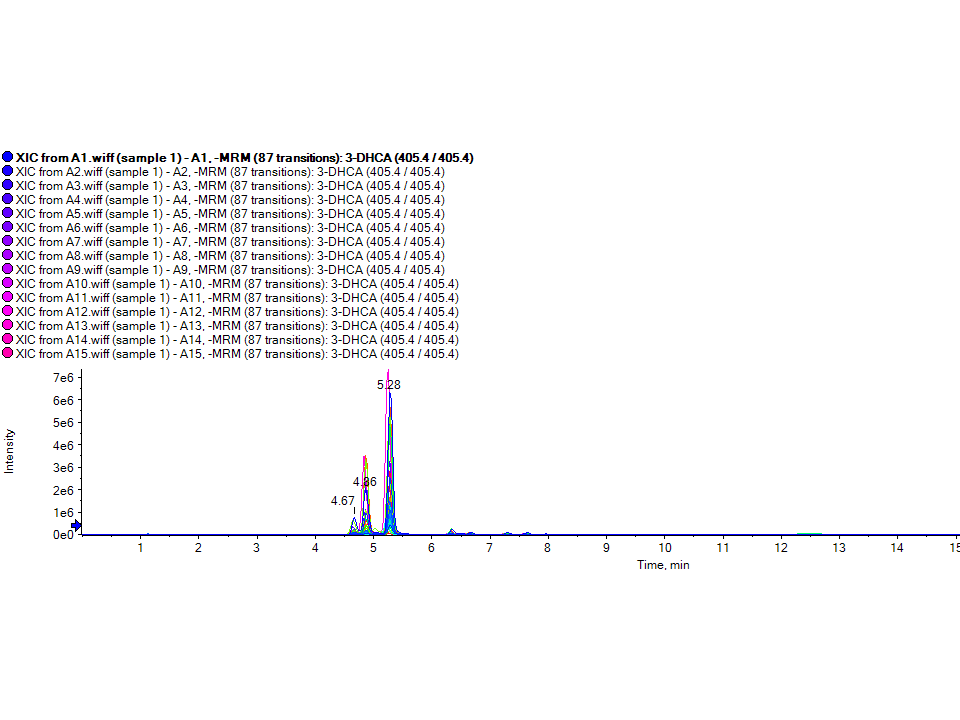

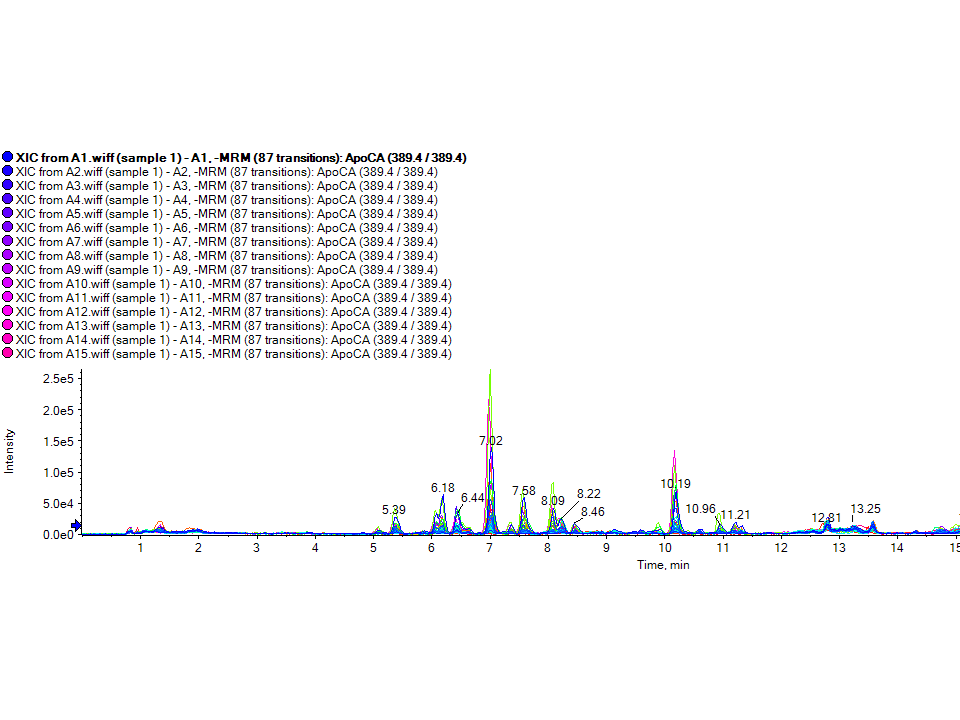

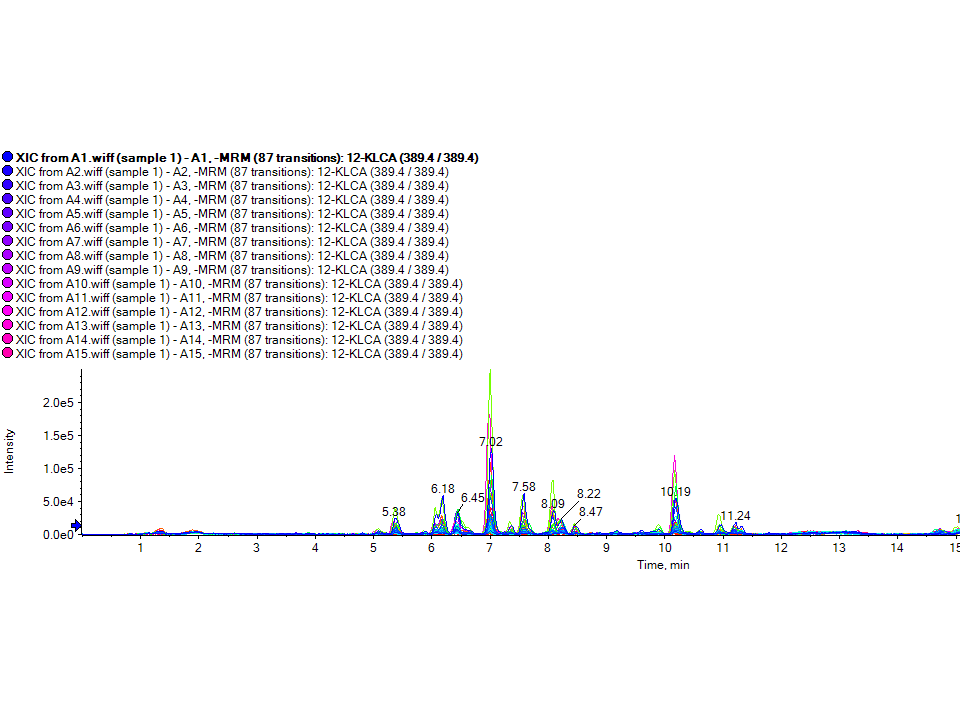

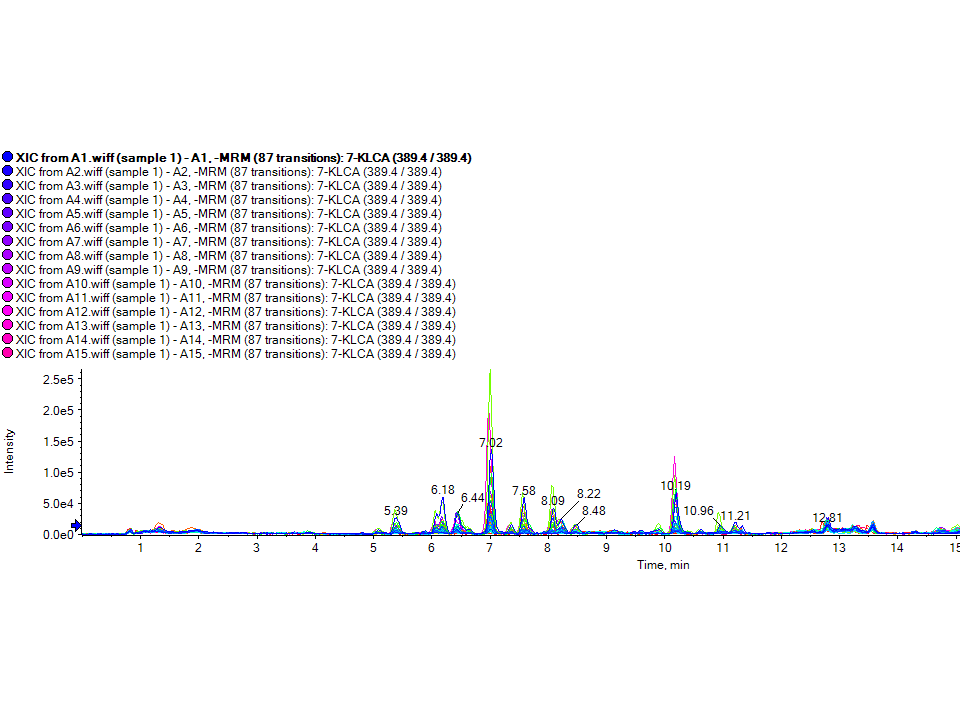

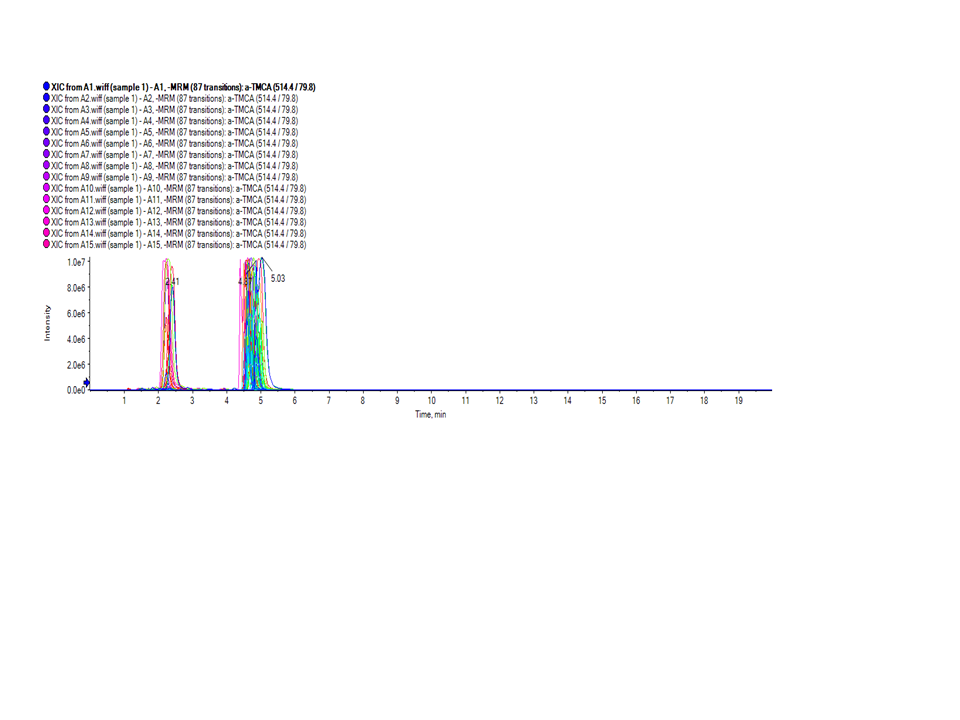

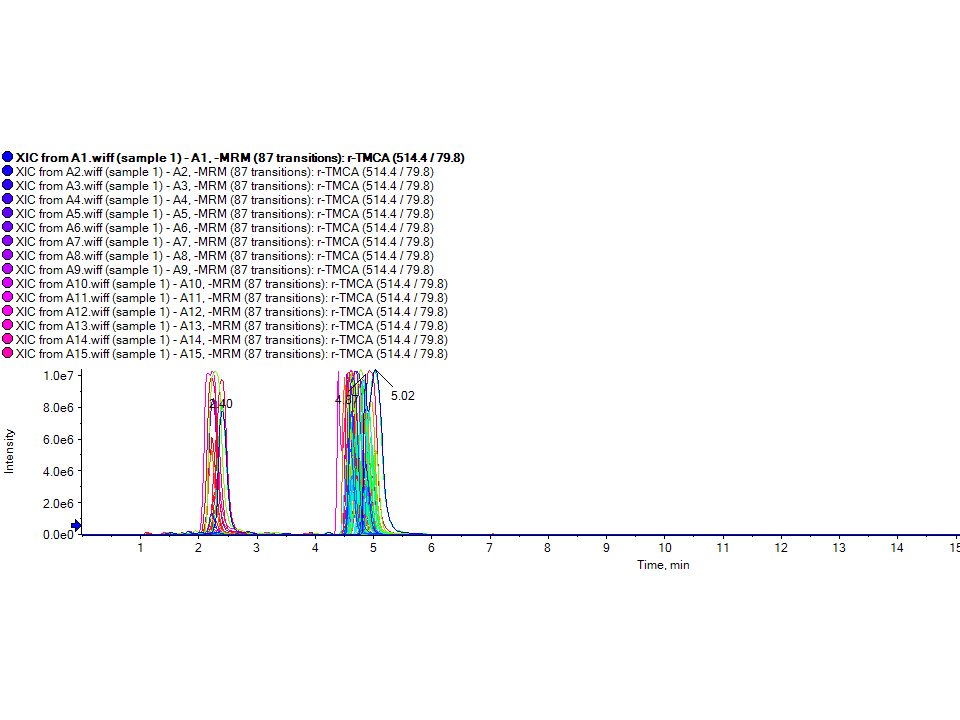

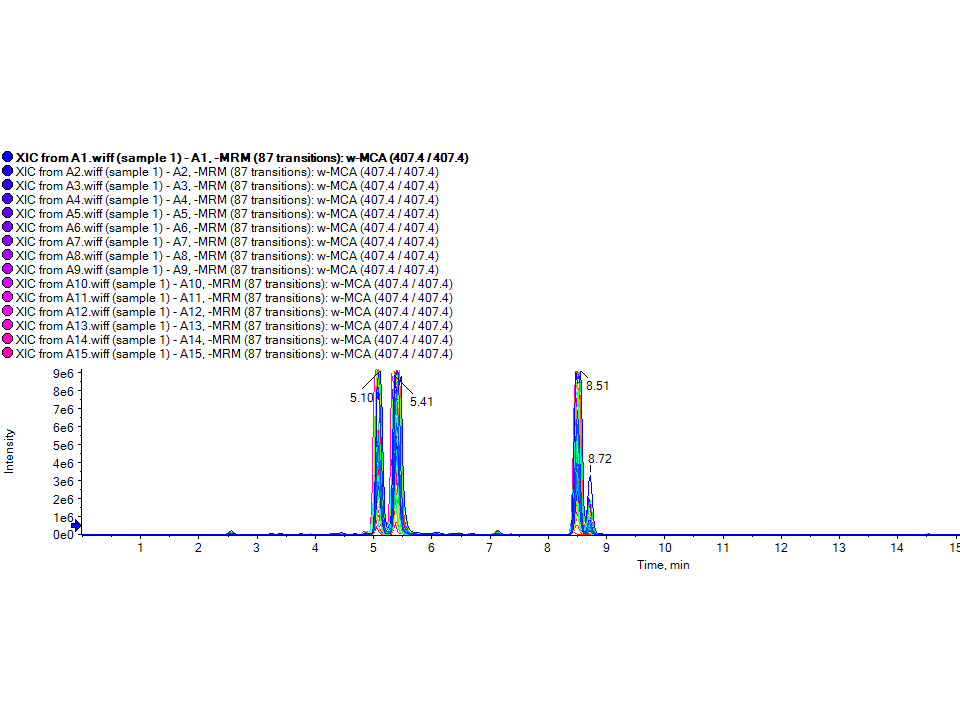

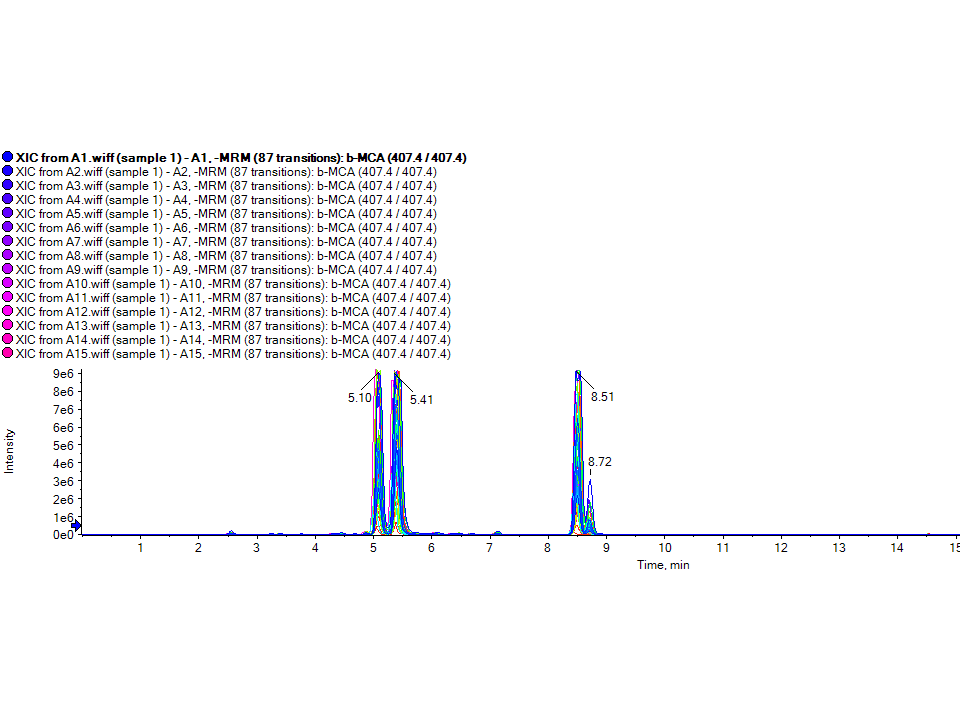

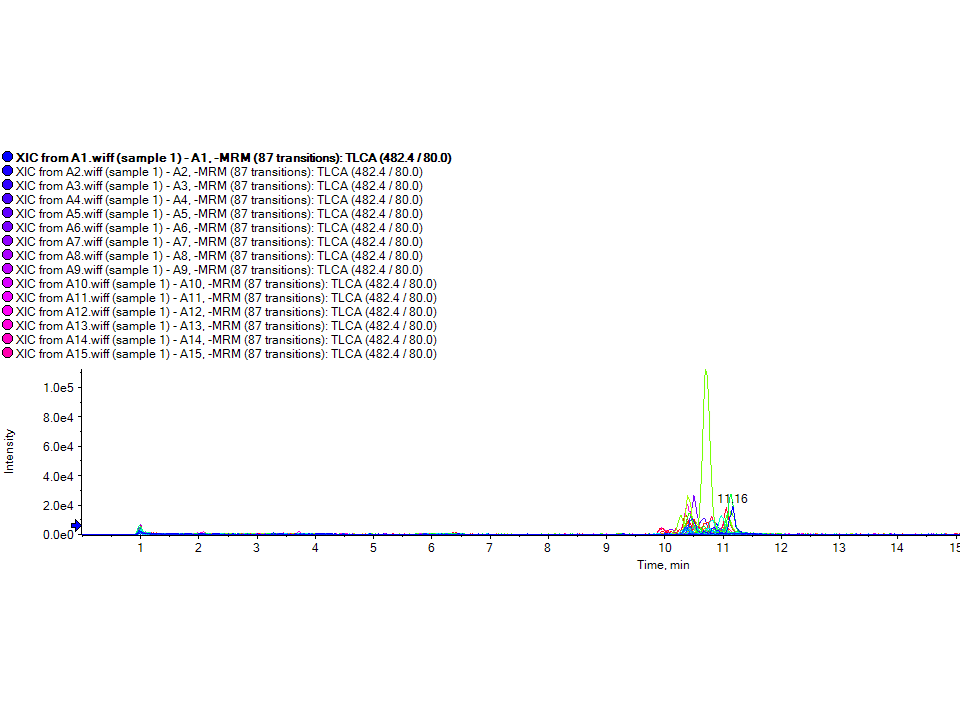

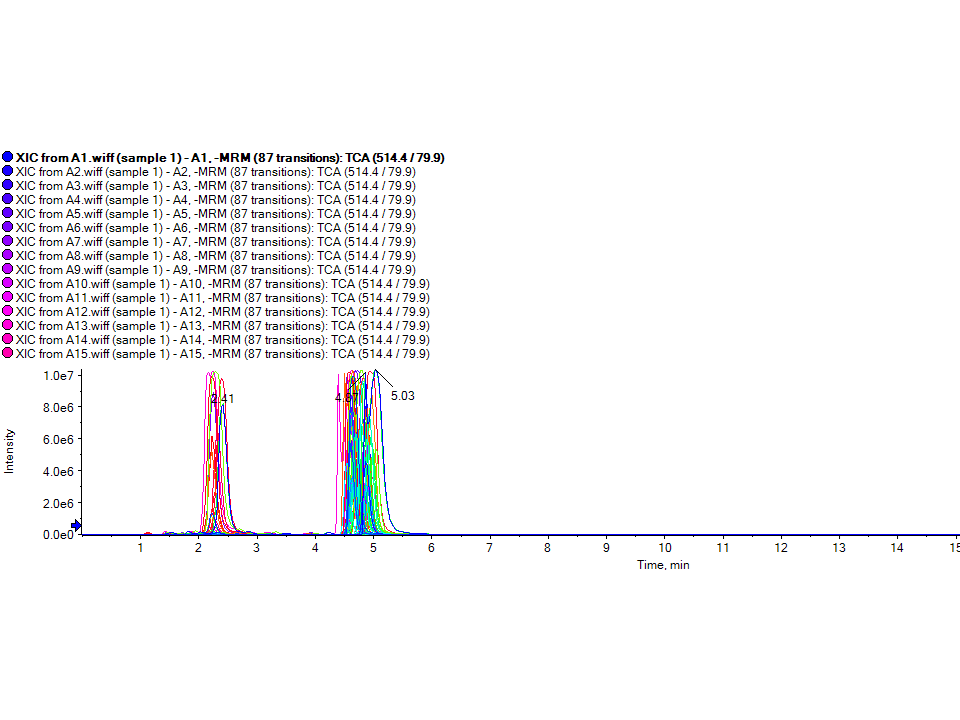

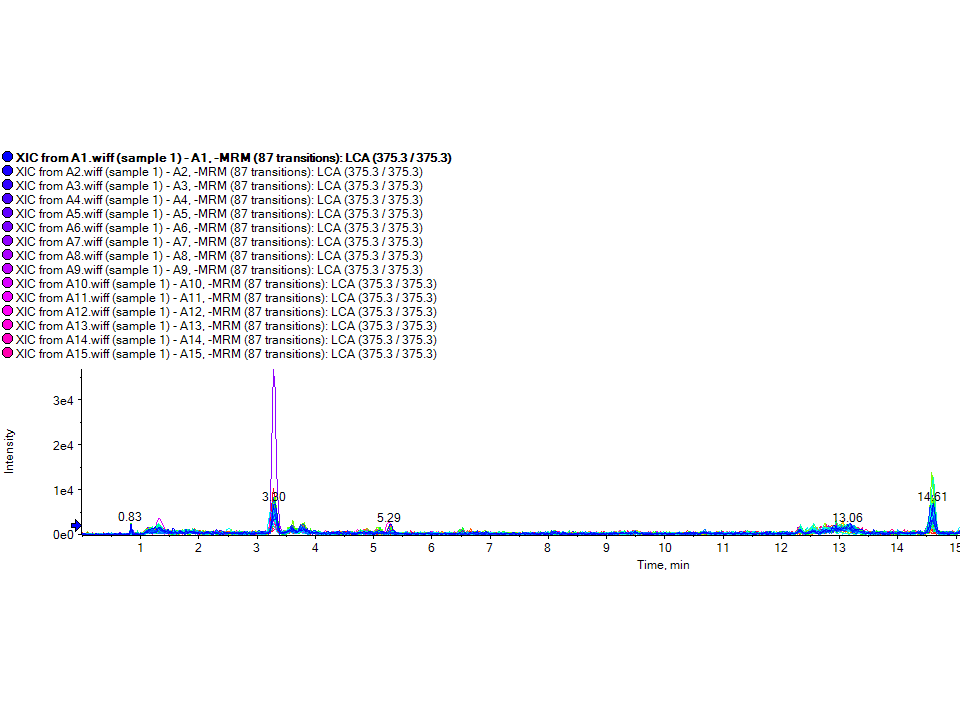

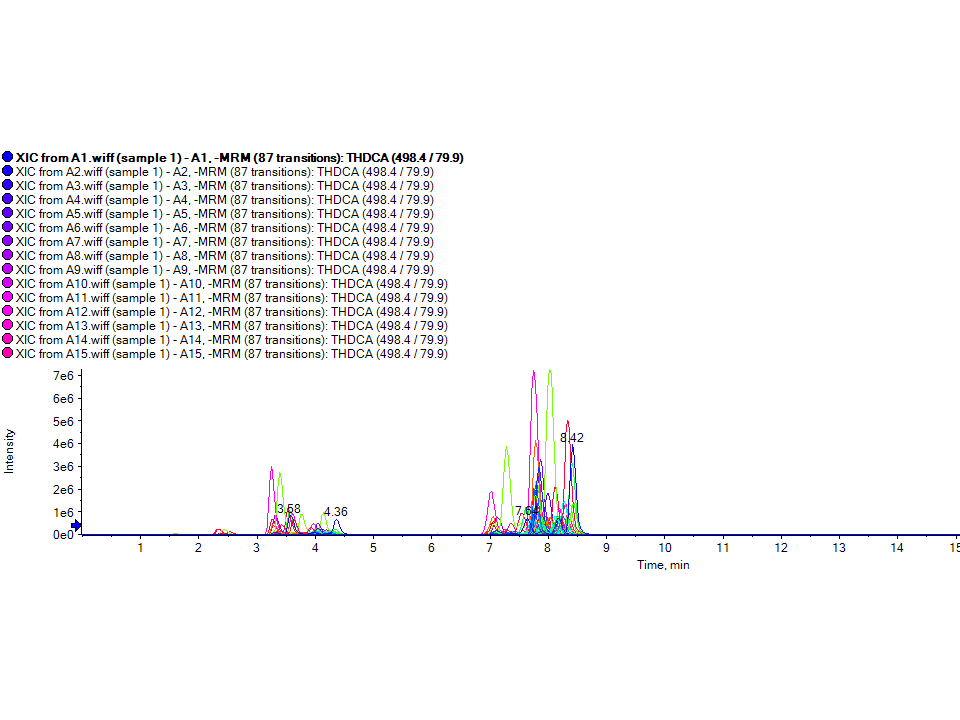

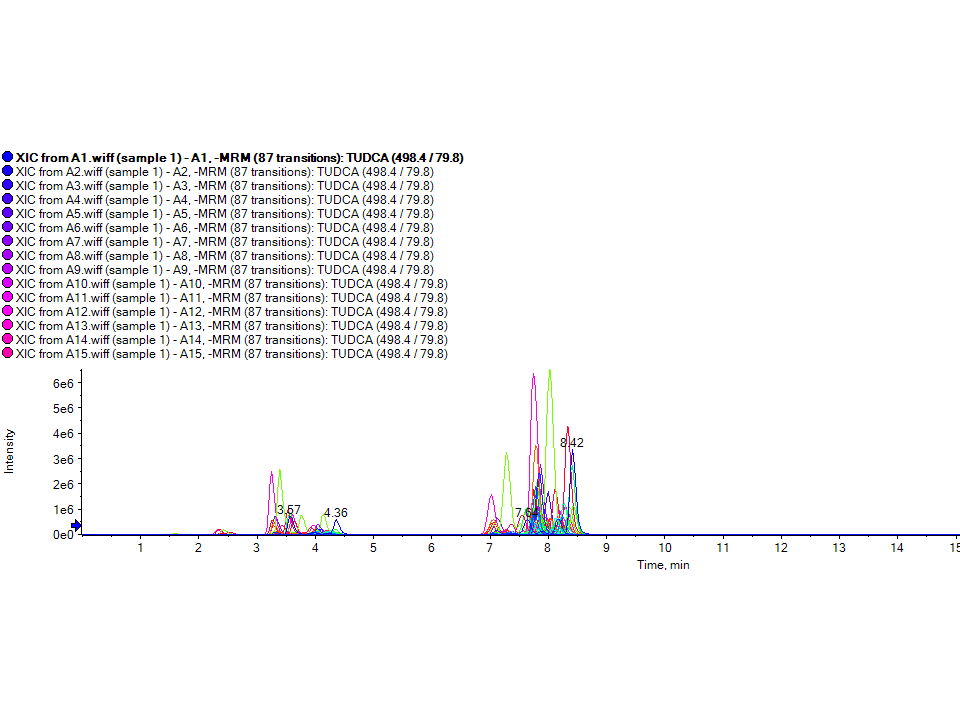

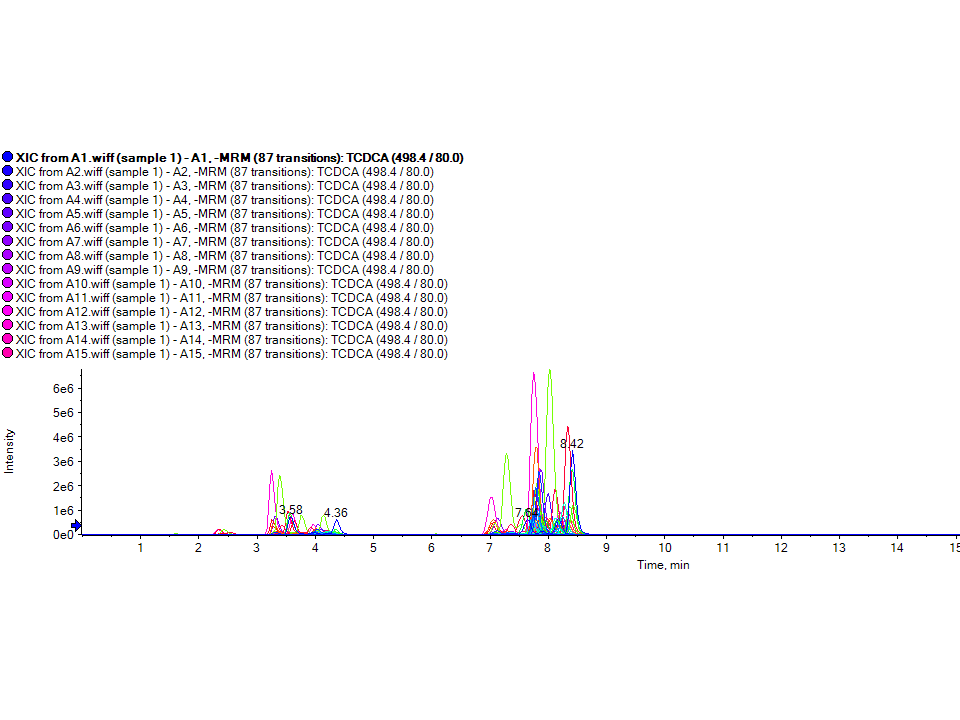

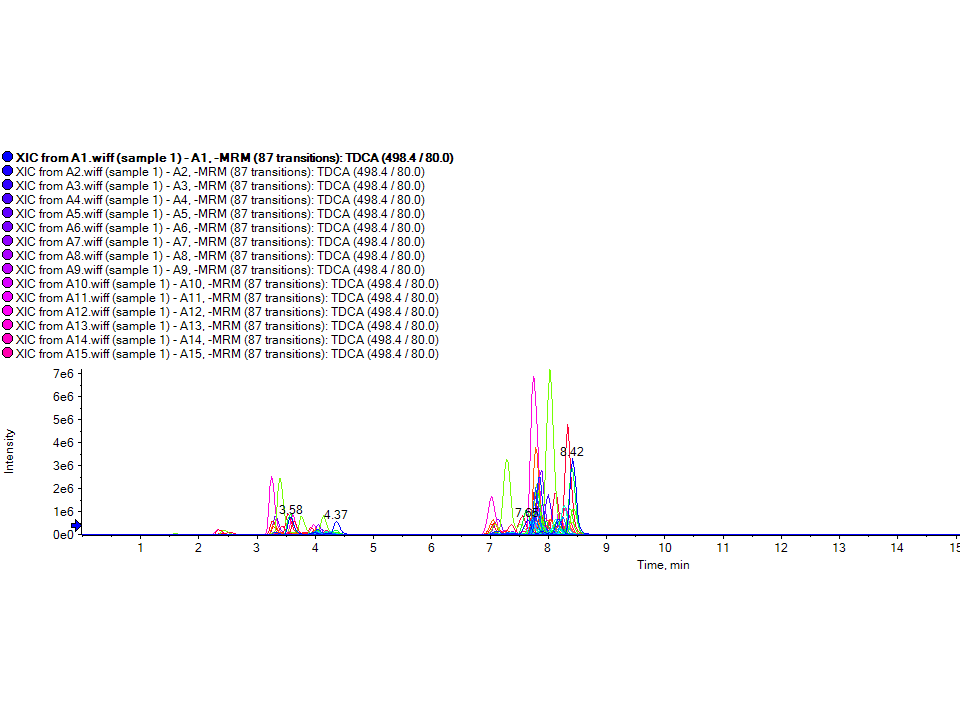

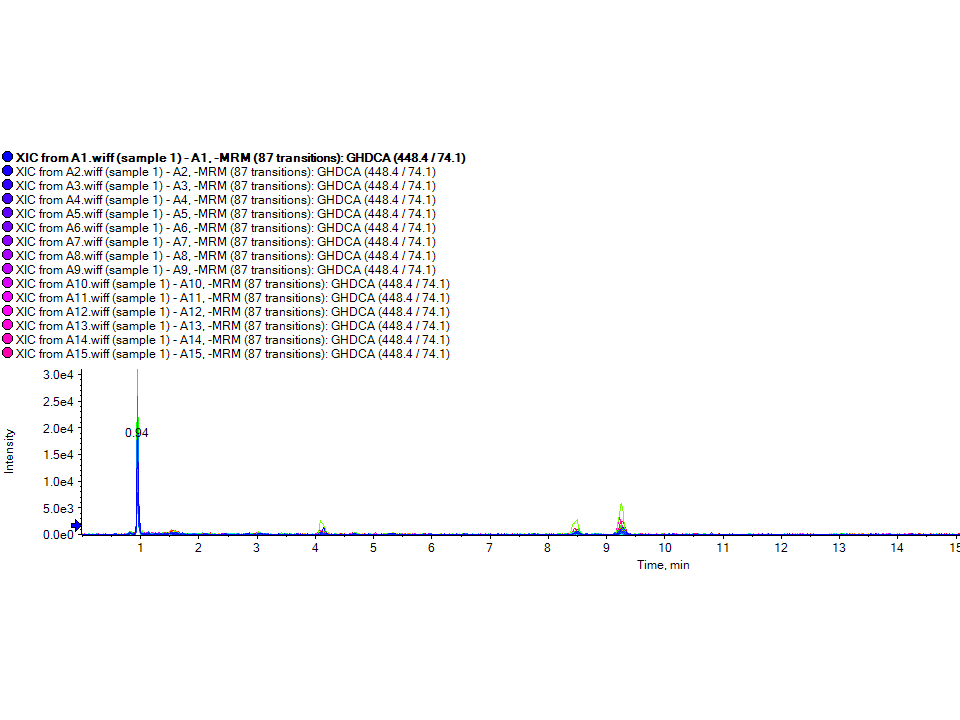

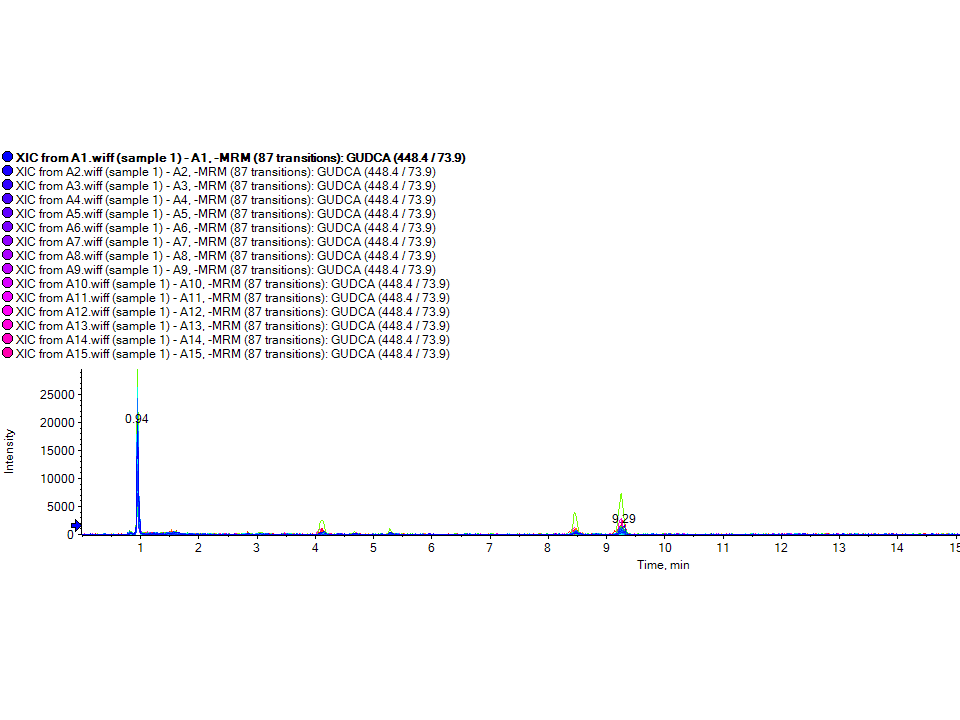

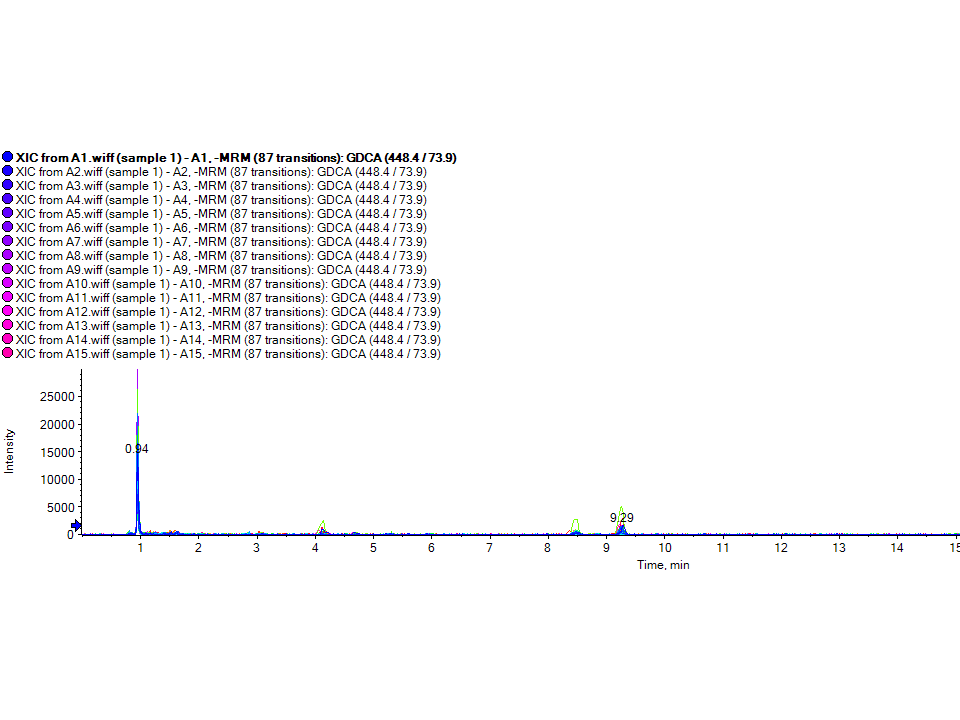

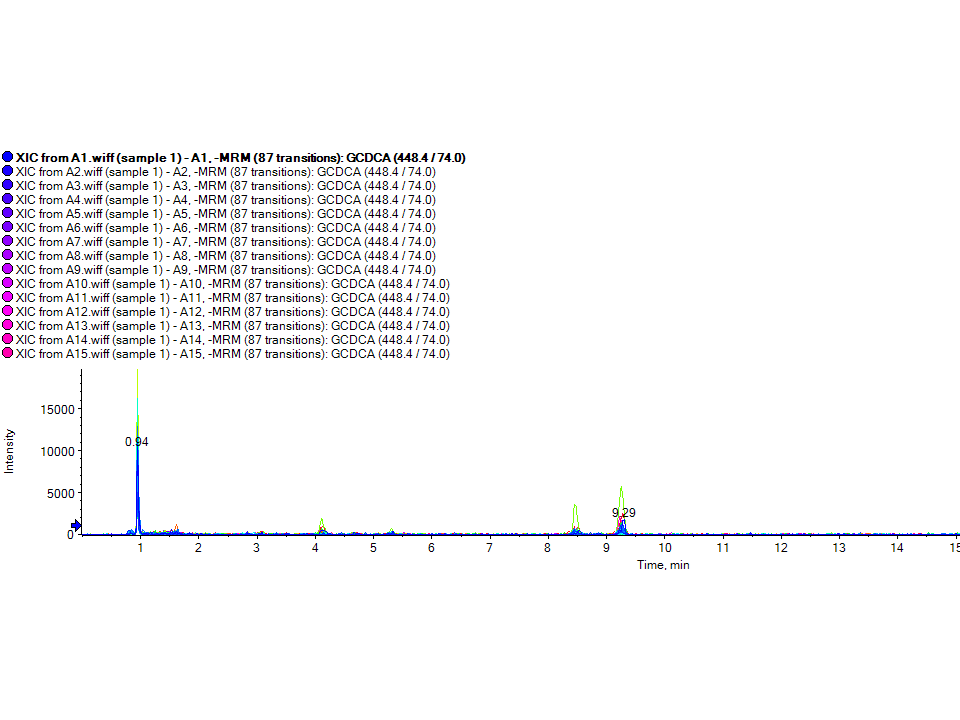

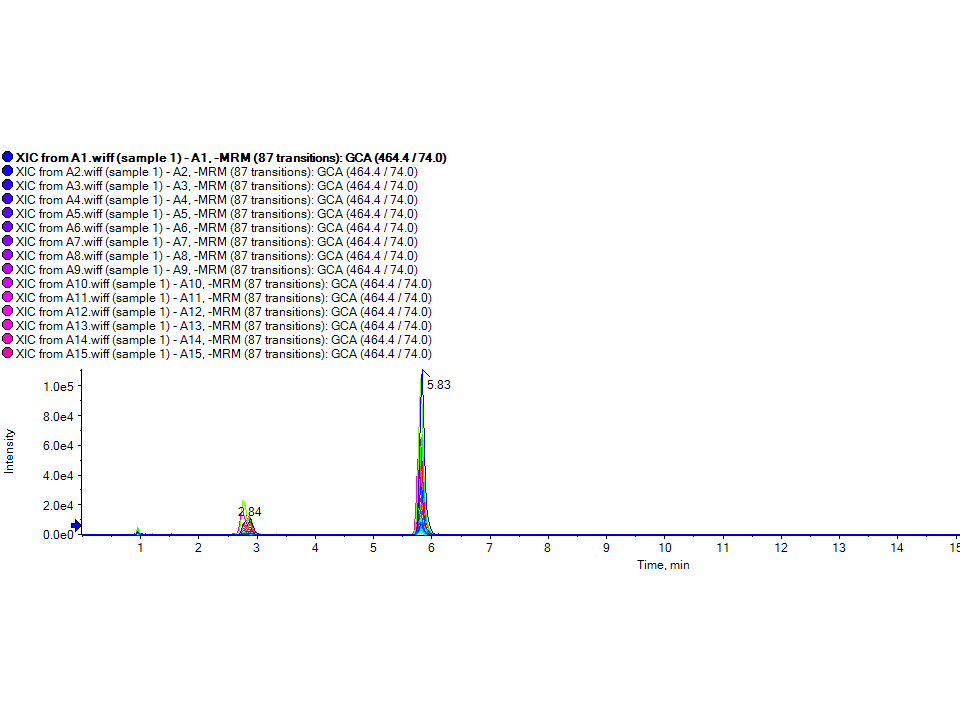

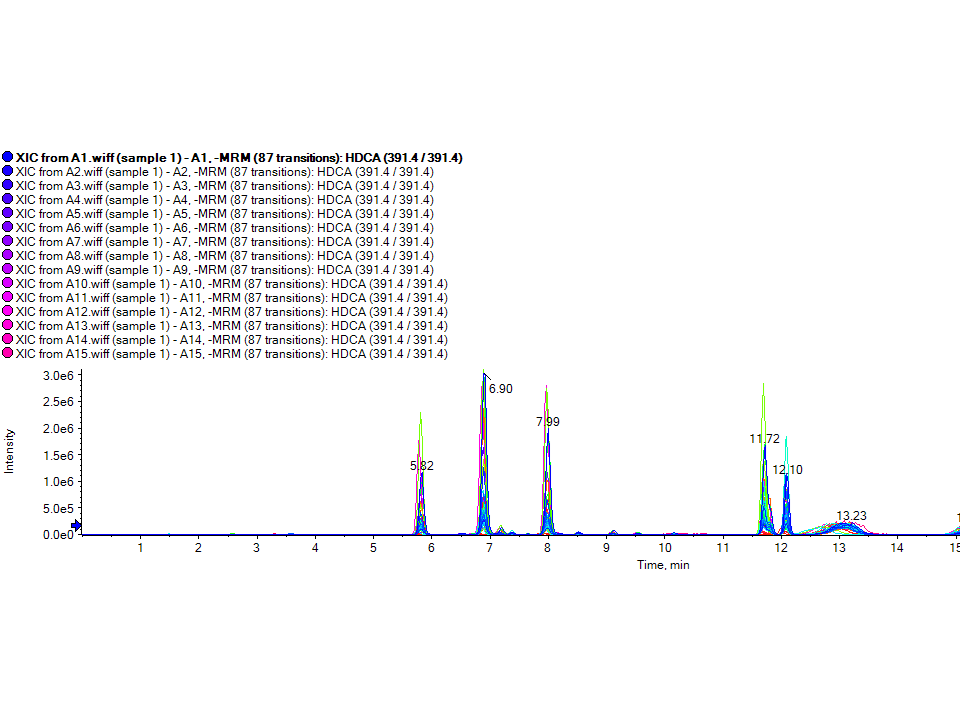

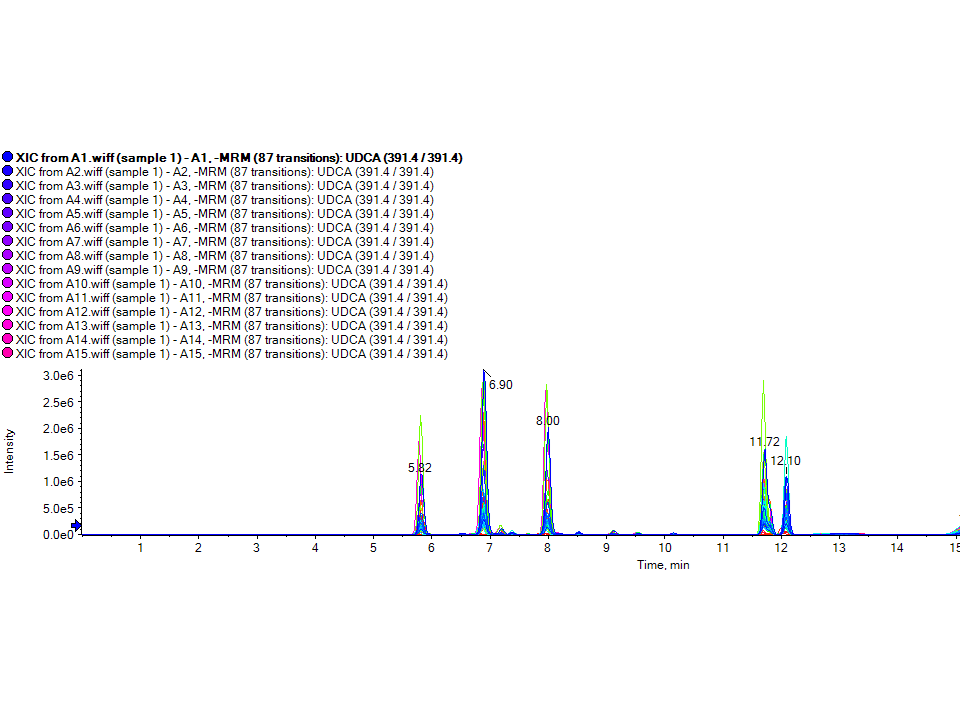

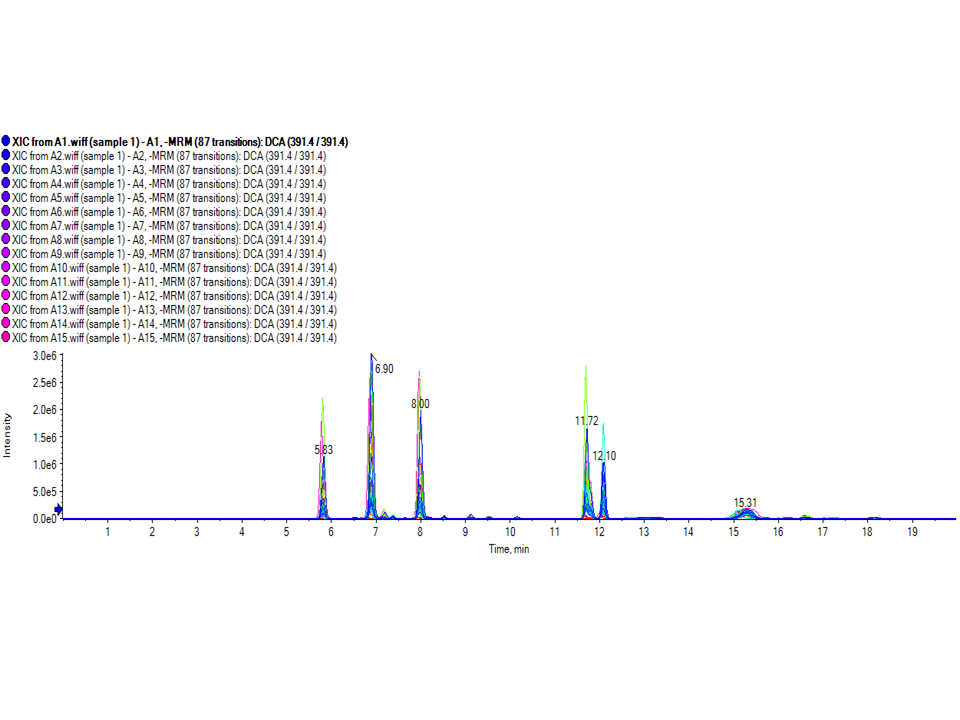

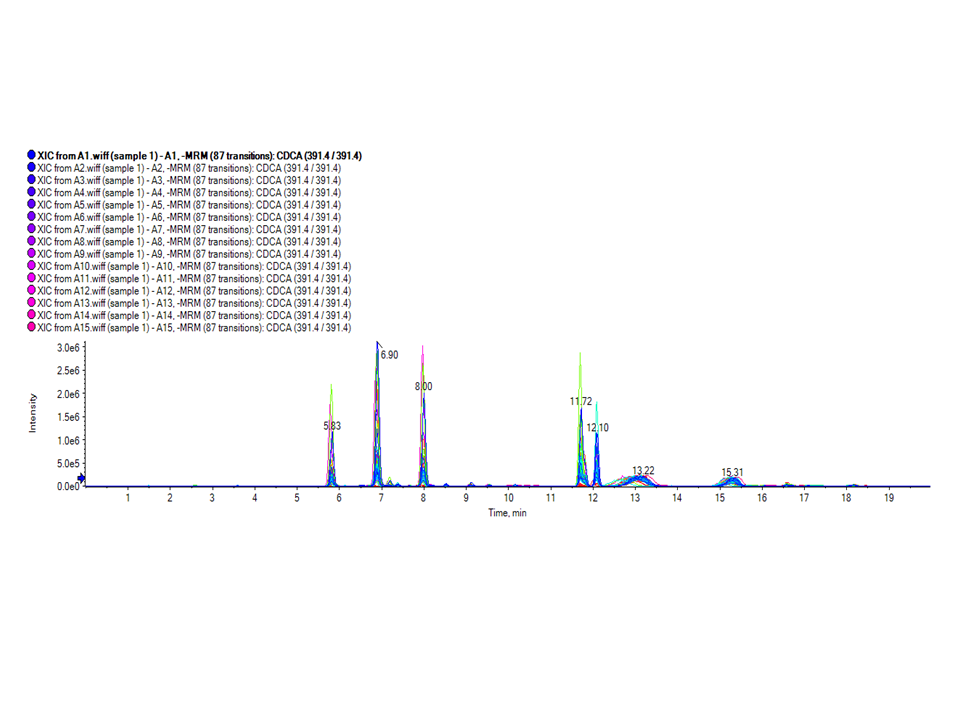

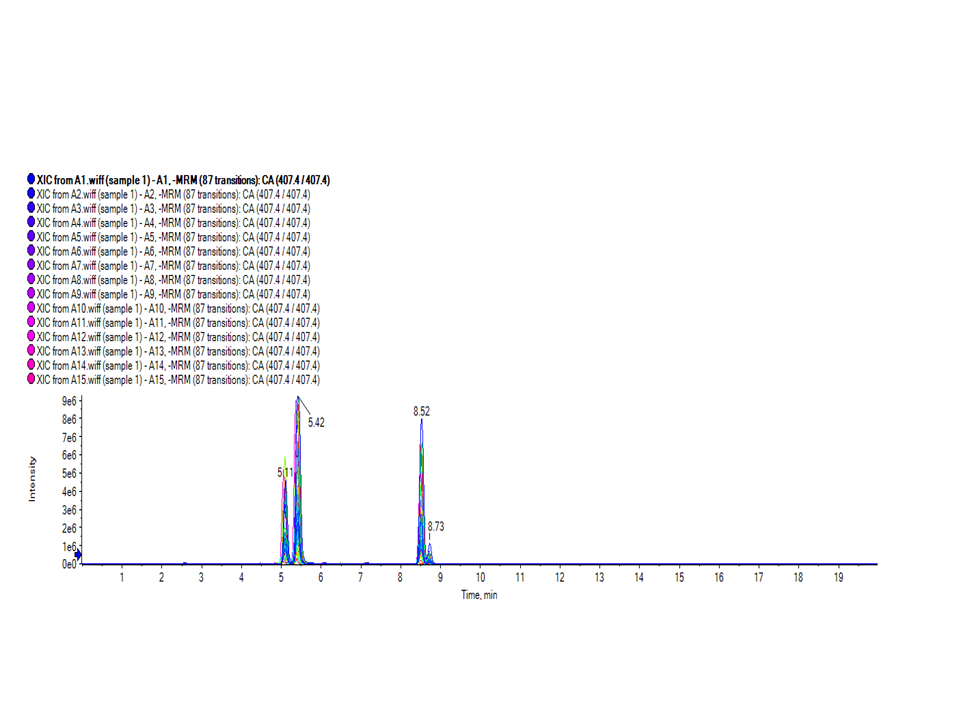

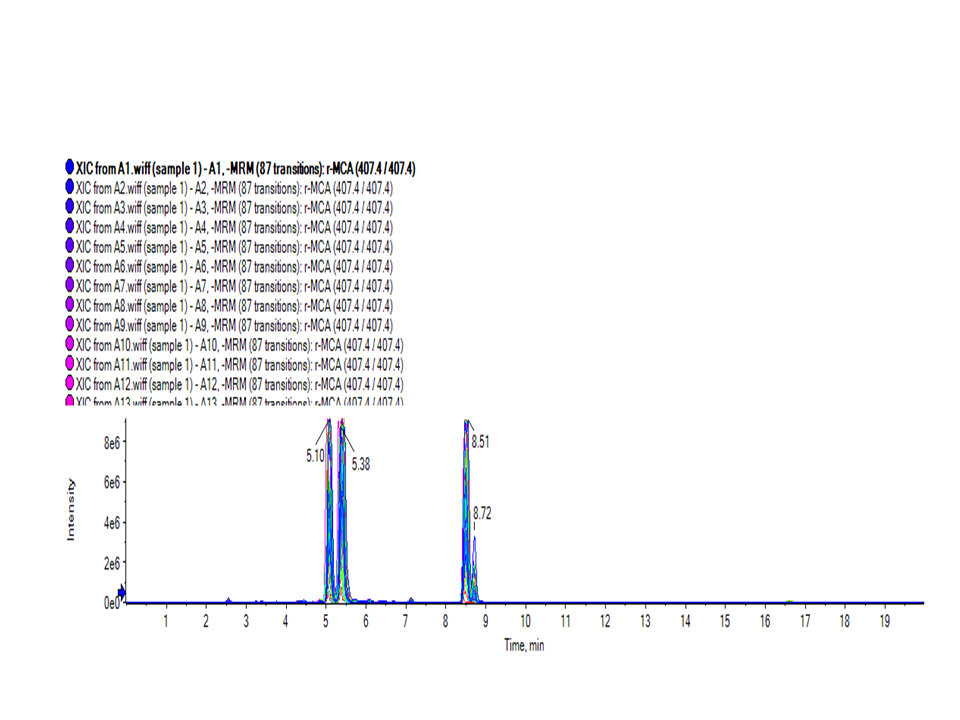

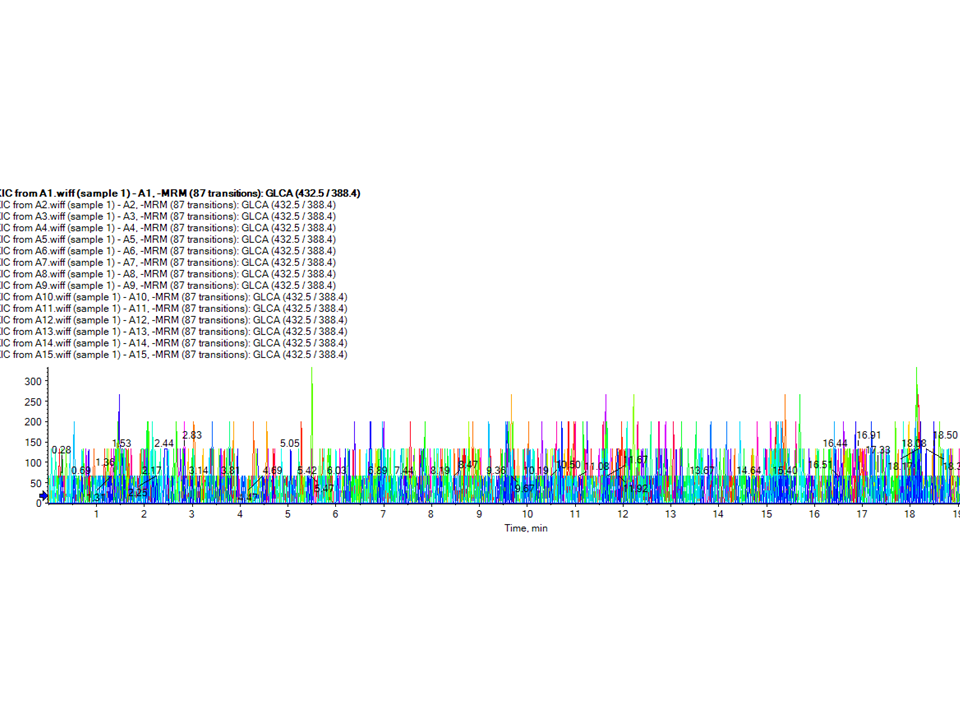

Supplement: Supplementary file 7 — Supplementary Information 7. [file 41598_2021_84694_MOESM7_ESM.docx]
