# Supplementary material for: Combined LC–MS/MS and 16S rDNA analysis on mice under high temperature and humidity and Herb Yinchen protection mechanism
Source: Sci Rep. 2021 Mar 3;11:5099. doi: 10.1038/s41598-021-84694-9 (PMC7930127; doi:10.1038/s41598-021-84694-9)

XIC from STD_C1.wiff (sample 1) - STD_C1, -MRM (87 transitions): CA (407.4 / 407.4)

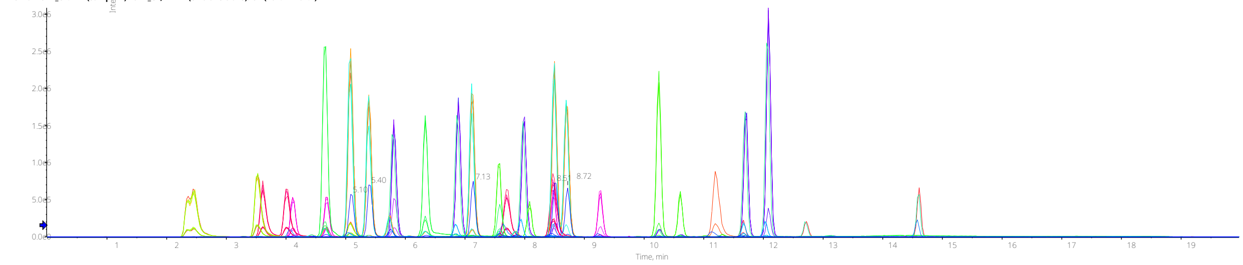

XIC from STD_C2.wiff (sample 1) - STD_C2, -MRM (87 transitions): CA (407.4 / 407.4)

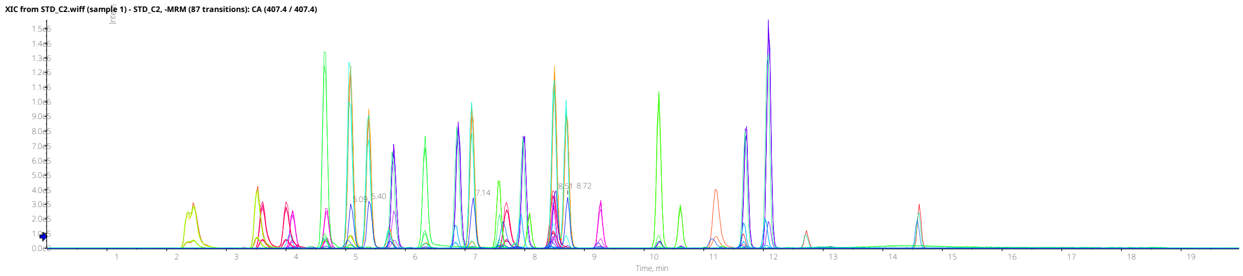

XIC from STD_C3.wiff (sample 1) - STD_C3, -MRM (87 transitions): CA (407.4 / 407.4)

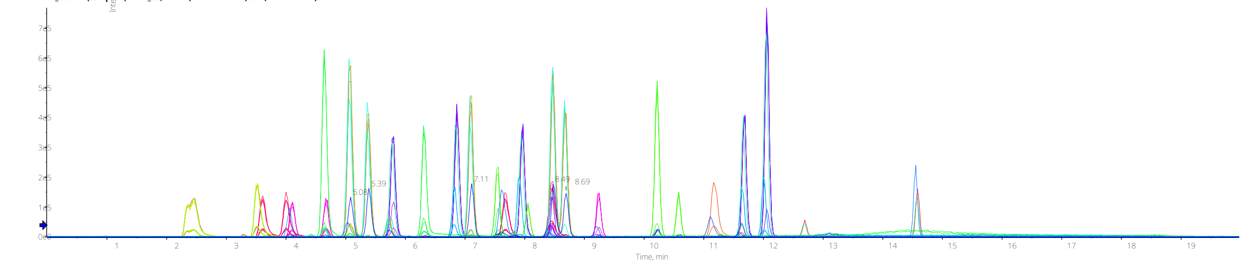

XIC from STD_C4.wiff (sample 1) - STD_C4, -MRM (87 transitions): CA (407.4 / 407.4)

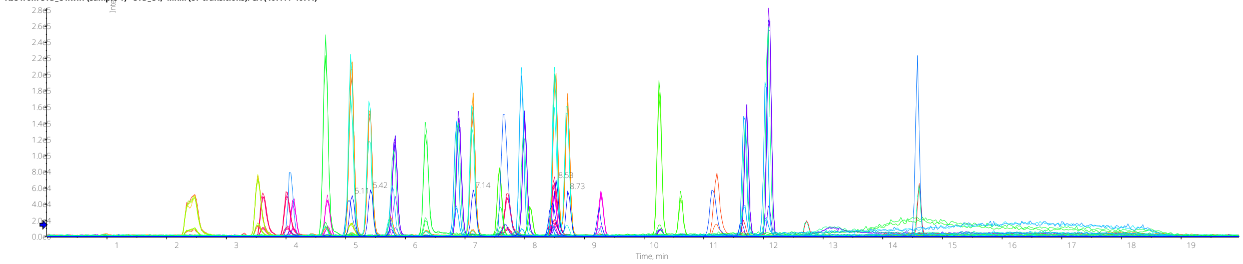

XIC from STD_C5.wiff (sample 1) - STD_C5, -MRM (87 transitions): CA (407.4 / 407.4)

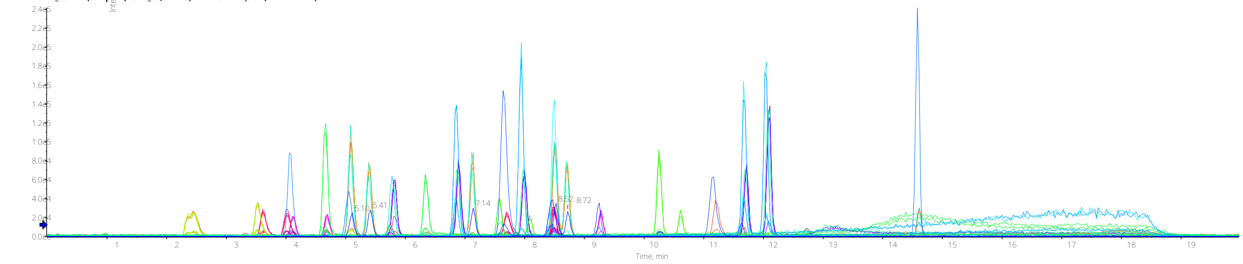

XIC from STD_C6.wiff (sample 1) - STD_C6, -MRM (87 transitions): CA (407.4 / 407.4)

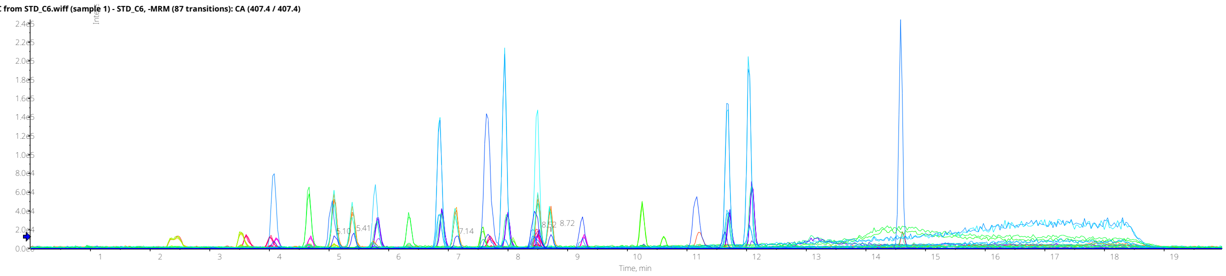

XIC from STD_C7.wiff (sample 1) - STD_C7, -MRM (87 transitions): CA (407.4 / 407.4)

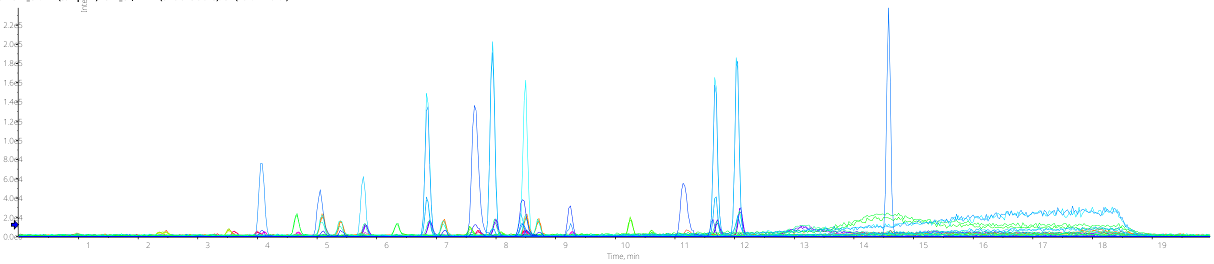

XIC from STD_C8.wiff (sample 1) - STD_C8, -MRM (87 transitions): CA (407.4 / 407.4)

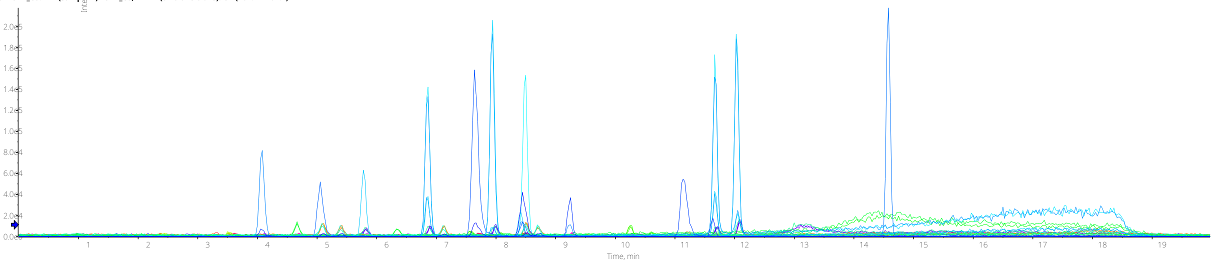

XIC from STD_C9.wiff (sample 1) - STD_C9, -MRM (87 transitions): CA (407.4 / 407.4)

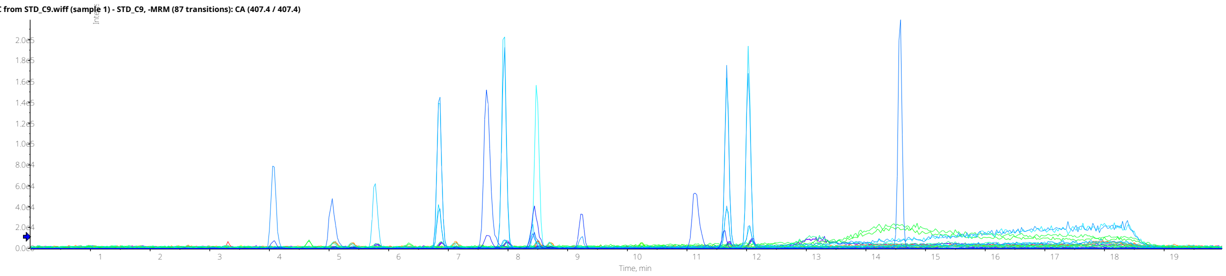

XIC from STD_C10.wiff (sample 1) - STD_C10, -MRM (87 transitions): CA (407.4 / 407.4)

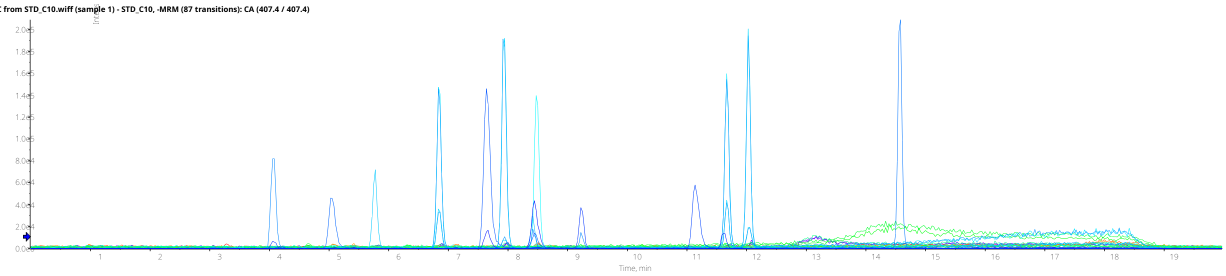

Supplement: Supplementary file 3 [file 41598_2021_84694_MOESM6_ESM.docx]
